# Characterization of Short Production Cycle and Nongenotoxic mRNA‐Based CAR‐T Cells Targeting CD19‐Positive and CD22‐Positive Malignancies

**DOI:** 10.1155/jimr/9452254

**Published:** 2026-07-30

**Authors:** Chenyun Zhang, Haizhou Liu, Hrithik Sangani, David Jin, Yen-Michael S. Hsu

**Affiliations:** ^1^ Tsinghua University School of Medicine, Beijing, China; ^2^ Department of Medicine, University of Pittsburgh, Pittsburgh, USA, pitt.edu; ^3^ Hong Kong Institute of Cell and Gene Therapy, Hong Kong, China; ^4^ UPMC Hillman Cancer Center, Pittsburgh, USA; ^5^ Department of Clinical Research, Gift of Life Marrow Registry, Boca Raton, USA; ^6^ Department of Medicine, Florida Atlantic University Charles E Schmidt College of Medicine, Boca Raton, USA, fau.edu

**Keywords:** cell therapy, chimeric antigen receptor, nonviral gene delivery

## Abstract

The chimeric antigen receptor (CAR) T cell therapy targeting cluster of differentiation 19 (CD19)‐positive malignancies has shown considerable efficacy in clinical settings. However, the potential genotoxicity of viral‐based CAR‐T therapy, their considerable manufacturing cycle and high costs, prompts questions about its safety and leads to limitations in clinical application. Aiming to explore a safe, efficient, and low‐cost mRNA‐based CAR‐T therapy, we characterize a nonviral, mRNA‐based approach utilizing a current good manufacturing practice (cGMP)‐compatible electroporation (EP) platform to generate anti‐CD19, anti‐CD22, and tandem anti‐CD19/CD22 CAR‐T products with a turn‐around time of less than 48 h. The protocol yields a more than 90% cellular viability with a CAR expression of exceeding 60% within the 24 h of transfection. The mRNA‐based CAR‐T products have a significantly enhanced T cell activation profile with CD69 upregulation, production of tumor necrosis factor‐alpha (TNF‐α), interleukin‐2 (IL‐2), and Granzyme B, and higher Ki67 expression level. Robust in vitro tumor neutralization by the mRNA‐based CAR‐T was observed compared to control. Interestingly, we have observed that the short‐term cell cryopreservation after EP resulted in a 50% reduction of cellular expansion. However, the cryopreservation and thaw did not have an observed negative impact on the in vitro tumor neutralization. Lastly, we have evaluated the metabolic activity of the mRNA‐based CAR‐T products, which has a clearly inducible and significant increase in basal respiration (by 60%) and a 2‐fold spare respiratory capacity (SRC) upon exposure with the antigen positive target cells. We believe this approach holds promise as a viable alternative that addresses the limitations of current CAR‐T therapies, offering a potential solution for future nonviral CAR‐T clinical application.

## 1. Introduction

The chimeric antigen receptor (CAR) T‐cell therapy is a revolutionary advancement in cancer immunotherapy, which has recently shown significant efficacy in studies across various cancer diseases. It involves creating an artificial receptor by combining the antigen‐binding region of a monoclonal antibody with the signaling domain of the T cell receptor (TCR) and potential costimulatory components [1, 2]. After introducing this synthetic receptor into T cells, it enables the effective binding of T cells to specific surface antigens on cancer cells. This specificity triggers T cell proliferation, cytotoxicity, and the release of cytokines. The remarkable efficacy of this cellular therapy lies in its ability to overcome the limitations of major histocompatibility complex (MHC) recognition in T cell‐mediated immune responses. By directly designing and manipulating the CAR structure, T cells can be engineered to specifically target and recognize designated cancer cells, unleashing intensive cellular immune responses mediated by T cells. Since the inception of the CAR‐T cell therapy concept, numerous studies have focused on research and improving the efficacy of its structural design. Eventually, designs predominantly featuring the coupling of cluster of differentiation (CD)3‐ζ with CD28 or 4‐1BB costimulatory domains have emerged [3–8]. In clinical settings, these designs have shown remarkable therapeutic effectiveness, mainly in patients with hematologic malignancies. As of now, various types of CAR‐T therapies have achieved significant clinical success, particularly in B cell acute lymphoblastic leukemia (B‐ALL) and relapsed/refractory lymphomas, where CD19, CD20, and CD22 serve as primary antigens [9–12]. Among early‐stage treatment, 80% patients have achieved complete response (CR), and the efficacy has been sustained in the long term, with a 12‐month event‐free survival rate reaching 50% [8, 13]. Beyond hematological malignancies, CAR‐T cell products are also under investigation for autoimmune rheumatic disorders, with favorable outcomes reported even in refractory/relapsed and heavily pre‐treated populations [14]. Based on the support from these promising preclinical and clinical data, the United States Food and Drug Administration (FDA) has approved a variety of CAR‐T therapies, including Abecma (idecabtagene vicleucel), Breyanzi (lisocabtagene maraleucel), Kymriah (tisagenlecleucel), Tecartus (brexucabtagene autoleucel), Yescarta (axicabtagene ciloleucel), Carvykti (ciltacabtagene autoleucel), and Aucatzyl (obecabtagene autoleucel). Despite the significant success of this therapy in CD19‐positive B‐ALL, it still has considerable limitations. First, for some patients with high tumor burden who have undergone various alternative cancer treatments, the responsiveness to CAR‐T therapy may not be ideal, often accompanied by relapse related to immune escape including the loss of CD19 antigen [15]. Second, CAR‐T cell therapy can be associated with both acute and long‐term toxicities. Among the acute complications, cytokine release syndrome (CRS) is a well‐recognized early‐onset inflammatory response, with endothelial injury recently implicated in its pathogenesis [16]. In addition to CRS, long‐term effects such as prolonged cytopenias and B‐cell aplasia may also impact patient outcomes [17]. In addition, the recent FDA announcement regarding the serious risk of T‐cell malignancy following BCMA‐directed or CD19‐directed autologous CAR T cell Immunotherapies has prompted ongoing evaluation of the long‐term safety profile of these therapies. While most acute toxicities, such as CRS and ICANS, are generally manageable in clinical practice [18], the potential for rare but serious late effects, including secondary T‐cell malignancies, underscores the importance of continued monitoring and the development of alternative platforms with potentially reduced genotoxic risk.

The widely used CAR‐T therapies currently employ viruses as vectors to introduce the CAR‐encoding construct into T cells, particularly using gammaretroviral and lentiviral vectors. However, the production process typically lasts 2–3 weeks. The extended time and complex production process [19] result in a long production cycle, high costs, and insufficient scale for CAR‐T cells produced with viruses, while the total upfront cost for CAR‐T therapy can reach a staggering $373,000–$711,884 [20] per patient. Currently, autologous CAR‐T therapy is commercially available only for patients with relapsed/refractory hematologic malignancies. It comes with a significant economic burden, which greatly limits its current application. This financial barrier is particularly pronounced in resource‐limited settings, where access to CAR‐T therapies remains extremely restricted due to the high costs of manufacturing, facility requirements, and specialized personnel training. Additionally, viral‐manufactured CAR‐T cells achieve stable and long‐term gene transfer and expression by integrating the gene into the genome, which may raise safety concerns and pose risks such as insertional oncogenesis. Another critical aspect is that viral vectors may encode epitopes that trigger immune responses in the host upon introduction [21]. CAR‐T cells manufactured using such methods often face the risk of immunogenicity, limiting their ability to fully exert their effects in vivo and reducing the duration of their effectiveness. Due to the numerous limitations associated with virus‐mediated CAR‐T cell production, there is an increasing demand to explore nonviral CAR‐T therapies. The goal is to reduce costs, shorten production cycles, scale up production, ensure safety while maintaining therapeutic potency, and ultimately expand its clinical application.

The manufacturing process of nonviral CAR‐T cells circumvents the use of viral vectors, where techniques, such as electroporation (EP), liposome‐based methods, and nanoparticles, are employed to create transient pores on the cell membrane, allowing the entry of exogenous genetic materials, including DNA, mRNA, and plasmid vectors, into the cells. Among these vectors, whether in the field of vaccines or cancer immunotherapy, RNA has increasingly broad applications [22–24]. One of the key features of introducing mRNA CAR into cells through a nonviral approach is that mRNA CAR can be rapidly synthesized and expressed in the cytoplasm without the need for the genomic integration process, thus naturally avoiding the risk of genotoxicity. Since mRNA located in the cytoplasm will not form a stable expression cell line, expression typically gradually diminishes after 2–4 cell cycles, creating a transiently transduced entity. This is why there is a growing belief that mRNA‐based CAR‐T represents a new path for CAR‐T therapy, enhancing safety significantly without significantly compromising efficacy while also offering the benefits of reduced time and cost.

## 2. Results

**Figure 1:**
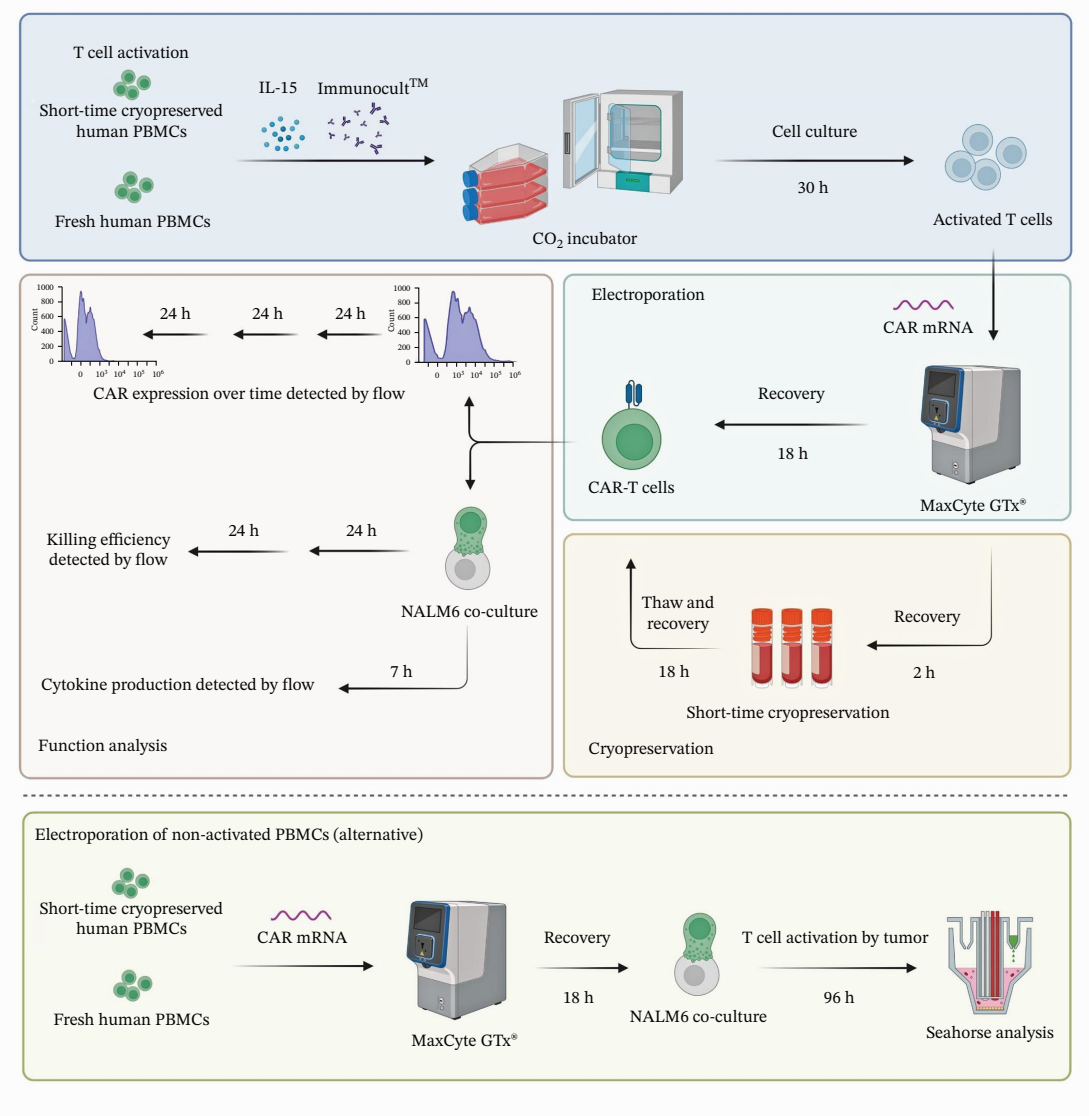
Optimized protocol for generating mRNA‐based CAR T using activated human PBMCs via Maxcyte GTx. Schematic depicting the generation and functional analysis of mRNA‐based CAR T cells using activated or nonactivated human PBMCs. CAR, chimeric antigen receptor; IL, interleukin; PBMC, peripheral blood mononuclear cell.

To optimize the protocol for generating mRNA‐based CAR‐T, we developed a 2‐day mRNA CAR‐T production protocol, utilizing three mRNA constructs encoding the CAR proteins with anti‐CD19, anti‐CD22, and anti‐CD19/CD22 specificities (Figure 1). In short, the human T cells or peripheral blood mononuclear cells (PBMCs) were activated with Immunocult (soluble anti‐CD3/CD28/CD2) and human interleukin (IL)‐15 for 30 h, followed by EP (Maxcyte ExPERT GTx system). Nonactivation protocol only requires a 18‐h resting period post‐EP. The mRNA CAR‐T cell products have shown a significant CD69 activation peak at around 30 h (Figure S1A), suggesting an optimal time for performing mRNA EP. The results of electroporating the human T cell line (Jurkat) with GFP mRNA concentrations ranging from 1 to 500 ng/µL demonstrated the high efficiency of mRNA EP using the Maxcyte GTx system (Figure S1B). Even at a low mRNA concentration of only 1 ng/µL, over 95% of the Jurkat cells exhibited GFP positivity. As the mRNA concentration increased, the fluorescence intensity in the Jurkat cells gradually enhanced, indicates a positive correlation between the number of mRNA‐encoding gene expressed by cells and the quantity of mRNA used for EP. However, the proportion of GFP‐positive cells reached a saturation point with the increasing mRNA concentration.

**Figure 2:**
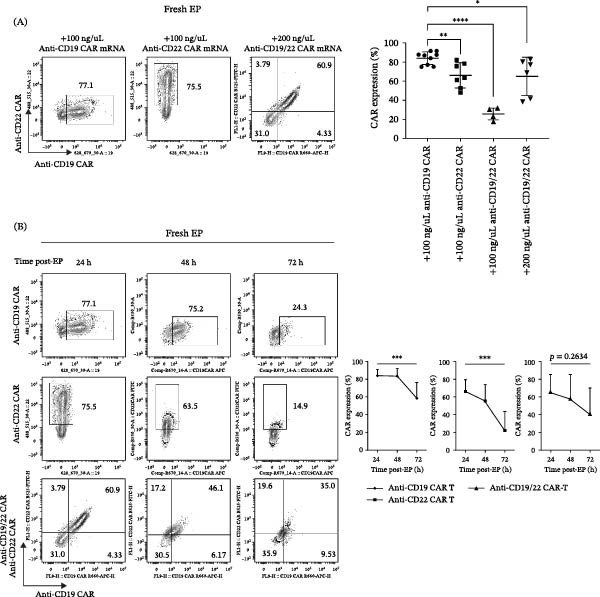
Electroporation of mRNA CAR produced transient CAR‐expressing T cells. (A) Activated human T cells from different donors (*n* = 3) were electroporated with 1 and 2 μg/10^6^ cell mRNA CAR via Maxcyte GTx using protocol expanded T cell 2. Multiple repeats were performed on each donor. CAR expression were detected by AF647‐CD19 and FITC‐CD22 on second day post‐EP. Expression rate of CD19/22 CAR‐T includes the population of both CD19‐CAR and CD22‐CAR monopositive cells in flow cytometry. (B) For fresh EP, CAR expression of different donors (*n* = 3) were detected successively from 24 to 72 h post‐EP. Data are representative of five independent experiments (anti‐CD19 CAR) or three independent experiments (anti‐CD22 CAR and anti‐CD19/22 CAR). CAR, chimeric antigen receptor; CD, cluster of differentiation; EP, electroporation.  ^∗^ *p*  < 0.05,  ^∗∗^ *p*  < 0.01,  ^∗∗∗^ *p*  < 0.001,  ^∗∗∗∗^ *p*  < 0.0001 by *t*‐test (A) and ANOVA (B). ns, nonsignificant.

The anti‐CD19, anti‐CD22, and anti‐CD19/CD22 CAR expressions were assessed. With the same mRNA content, the expression rate of anti‐CD19/22 is significantly lower (*p* < 0.0001) than that of the single‐target CARs. However, when the mRNA amount of anti‐CD19/22 CAR mRNA is increased to 200 ng/uL, its relative expression level becomes comparable to the other two CARs (Figure 2A). EP of 100 ng/uL of anti‐CD19 and anti‐CD22 CAR mRNA and 200 ng/uL of CD19/22 CAR mRNA produced 84.1%, 65.9%, and 64.9% of CAR expression, respectively (Figure 2A). To further evaluate the kinetics of mRNA‐based CAR expression, we tracked the expression rates of three CARs within the first 72 h post‐EP (Figures 2B and S2A,B). The results indicate that expression levels of three types of CAR T cell decrease to 58.4% (*p* < 0.001), 21.8% (*p* < 0.001), and 39.9% (*p* = 0.2634) for CD19, CD22, and CD19/22, respectively, on the third day after EP.

**Figure 3:**
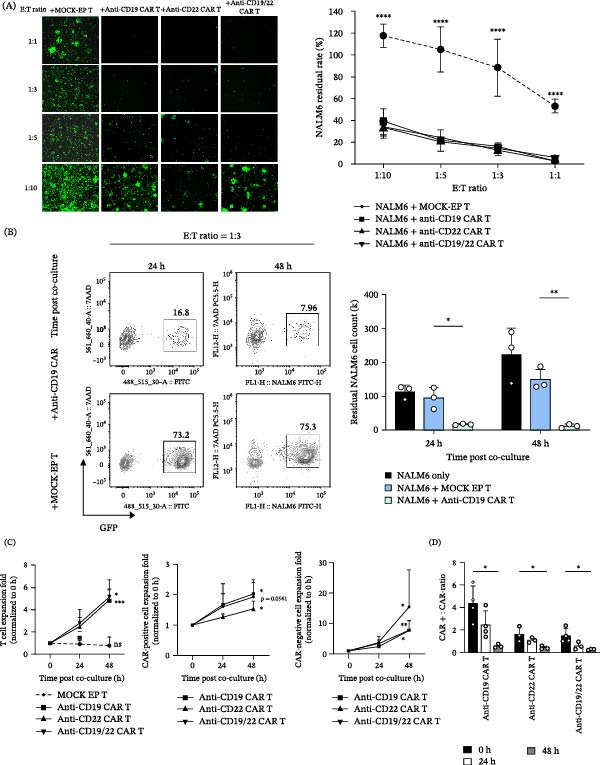
The mRNA‐based CAR‐T cells generated through electroporation exhibited robust tumor‐killing activity in vitro. (A) Fresh EP CAR‐T cells from different donors (*n* = 3) were co‐cultured with ZsGreen‐labeled NALM6 at an E:T ratio of 1:1 (50k + 50k), 1:3 (25k + 75k), 1:5 (25k + 125k), and 1:10 (25k + 250k). Effector cell count was normalized based on the actual CAR^+^ cells. Fluorescence pictures were taken after 24 h of co‐culture. Residual NALM6 cell count were measured by cell counting and flow, and residual rate was calculated by comparing to the NALM6 only groups. Data are representative of four independent experiments. (B) At an E:T ratio of 1:3, T cells and NALM6 cells were co‐cultured for 48 h, residual NALM6 cell count were measured by flow. Data are representative of three independent experiments. (C, D) For CAR‐T and MOCK cells generated from Donor 1, T cell expansion fold and ratio of CAR‐positive and negative T cells were measured by flow. Data are representative of four independent experiments. CAR, chimeric antigen receptor; CD, cluster of differentiation; EP, electroporation.  ^∗^ *p*  < 0.05,  ^∗∗^ *p*  < 0.01,  ^∗∗∗^ *p*  < 0.001,  ^∗∗∗∗^ *p*  < 0.0001 by *t*‐test (A, B) and ANOVA (C, D). ns, nonsignificant.

To examine the potency of the mRNA‐based CAR‐T cells to exert target‐specific cytotoxic effects, we co‐cultured CAR cells and MOCK‐EP cells separately with ZsGreen‐labeled NALM6 cells, expressing both CD19 and CD22 molecules on the cell surface [25, 26], at various effector‐to‐target (E:T) ratios. At an E:T ratio of 1:1, 1:3, 1:5, and 1:10, anti‐CD19 CAR‐T cells showed an inhibitory impact of 96.70% (*p* < 0.001), 85.24% (*p* < 0.05), 79.06% (*p* < 0.01), and 62.20% (*p* < 0.01) on NALM6 cells growth within 24 h (Figure 3A) compared to NALM6 only group, while anti‐CD22 and anti‐CD19/22 CAR‐T cells exhibit similar tumor‐killing capability. Compared to these mRNA‐based CAR‐T, MOCK‐EP T only exhibited limited NALM6 inhibitory effect (*p* < 0.0001). At an E:T ratio of 1:3 and 48 h post co‐culture, we observed a low residual cell count (*p* < 0.01) of NALM6 cells co‐cultured with anti‐CD19 CAR‐T cells, while the viable cell count of NALM6 cells co‐cultured with MOCK‐EP T cells continued to increase (Figure 3B). Also at the same E:T ratio, three types of CAR‐T cells exhibited a nearly five‐fold proliferation (*p* < 0.05, *p* < 0.001, and *p* < 0.001, respectively) within 48 h (Figure 3C). When focusing on the CAR‐positive and CAR‐negative populations within total T cells, we observed that these two cell populations proliferated at different rates during co‐culture. In the presence of target cells, the CAR expression ratio significantly decreased from initial values of 4.35–0.56, 1.61–0.43, and 1.47–0.27, respectively, for anti‐CD19, anti‐CD22, and anti‐CD19/22 CAR expression detected (Figure 3D).

To confirm whether this tumor suppression effect is CAR‐specific, we further expanded these CAR T cells in cell culture for 96 h post‐EP to reduce the expression rate of the CAR. Then, at 4 days post‐EP, we co‐cultured them with NALM6 cells for 24 h, and it was observed that regardless of the E:T ratio, the effects of the three CAR‐T cells are not significantly different from those of MOCK‐EP T (Figure S3A). To confer the specificity of CAR‐mediated tumor neutralization, various mRNA‐based CAR‐T did not show cytotoxicity with MDA‐MB‐231 cells which are negative for CD19 or CD22 expression (Figure S3B). This collectively indicates that these mRNA‐based CAR‐T cells exhibit tumor suppression effects that are both CAR‐specific and tumor‐specific.

**Figure 4:**
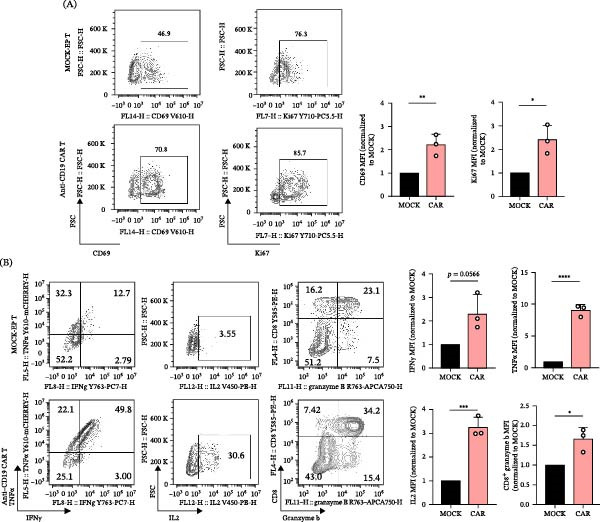
mRNA‐based CAR‐T cells generated through electroporation exhibited a distinct immunophenotype and cytokine secretion level in terms of cell cytotoxicity in vitro. Anti‐CD19 CAR T cells generated from different donors (*n* = 3) and NALM6 cells were co‐cultured at an E:T ratio of 1:1 24 h post‐EP, CD69 and Ki67 expression (A) and intracellular production of cytokines IFN‐γ, TNF‐α, IL2, and Granzyme B (B) were measured by flow after 7 h of co‐culture. Data are representative of three independent experiments. CAR, chimeric antigen receptor; CD, cluster of differentiation; EP, electroporation; FSC, forward scatter; IFN, interferon; IL, interleukin; MFI, mean fluorescence intensity; TNF, tumor necrosis factor.  ^∗^ *p*  < 0.05,  ^∗∗^ *p*  < 0.01,  ^∗∗∗^ *p*  < 0.001,  ^∗∗∗∗^ *p*  < 0.0001 by *t*‐test (A, B). ns, nonsignificant.

Next, we explore the immunophenotypic profile and the T‐cell activation‐induced cytokine secretion level of the mRNA‐based CAR‐T upon the exposure of the target cells. To confirm the proportions of T cell subgroups, we performed flow cytometry before and after 7, 24, and 48 h of NALM6 co‐culture. CD4, CD8, and CD69 were used as markers for helper T cells, cytotoxic T cells, and activated T cells, respectively. The immunophenotypic analyses showed that the expansion rate of CD8‐positive cells in anti‐CD19 CAR‐T was significantly faster than that in MOCK‐EP T (Figure S4), with the number of cytotoxic T cells being 1.16, 1.82, and 2.21 times higher than that of the control group at 7, 24, and 48 h, respectively. Similarly, the CD69 expression measured on CAR‐positive cells was more than two‐fold (*p* < 0.01) higher than MOCK‐EP T cell control group, while the Ki67 expression was 2.41‐fold higher (*p* < 0.01) (Figure 4A), indicating that the entire cell population tended to exhibit a higher level of activation and was actively proliferating. To validate whether our mRNA‐based CAR‐T cells exhibit enhanced cytokine production capacity, we used intracellular staining flow cytometry to detect the production levels of IFN‐γ, tumor necrosis factor‐alpha (TNF‐α), IL‐2, and Granzyme B in CAR cells and MOCK‐EP T cells. When co‐cultured with NALM6 cells, mRNA CAR‐T showed a significantly higher intracellular TNF‐α, IL‐2, and Granzyme B production (in CD8‐positive T cells) compared to MOCK‐EP control (Figure 4B).

**Figure 5:**
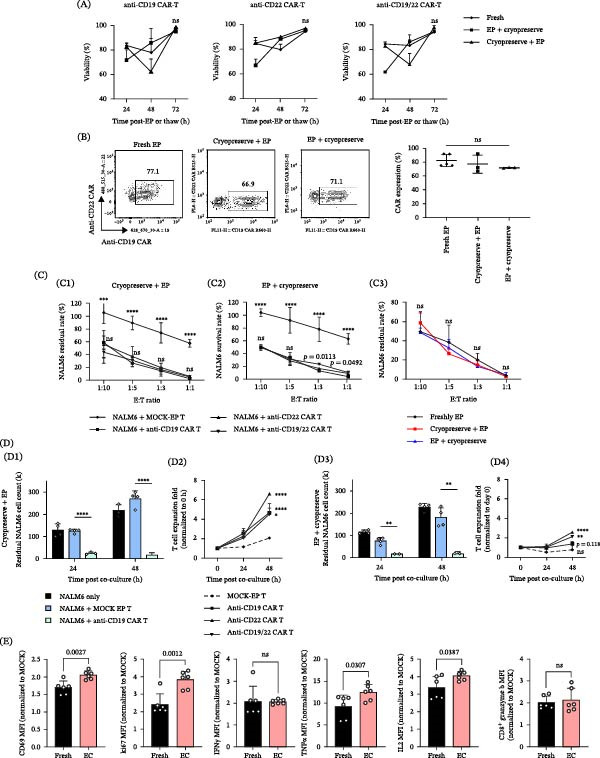
Short‐term cell cryopreservation before and after electroporation does not significantly impact the anti‐tumor functionality. (A) PBMCs from Donor 1 were cryopreserved for 2 weeks before (cryopreserve + EP) and after EP (EP + cryopreserve). Cell viability were measured by trypan blue successively from 24 to 72 h post‐EP or post‐thaw. Data are representative of three independent experiments. (B) CAR expression in different cell conditions were detected on second day post‐EP or post‐thaw. Data are representative of five independent experiments (fresh EP) or three independent experiments (EP + cryopreserve and cryopreserve + EP). (C) CAR T cells under various conditions were co‐cultured with NALM6, NALM6 residual rate were measured after 24 h of co‐culture. Killing curve of anti‐CD19 CAR‐T cells under different condition were compared. Data are representative of four independent experiments. (D) At E:T ratio of 1:3, T cells and NALM6 cells were co‐cultured for 48 h, residual NALM6 cell count and T cell expansion fold were measured by flow. Data are representative of four independent experiments. (E) For anti‐CD19 CAR T cells cryopreserved after EP, CD69 and Ki67 and intracellular production of cytokines IFN‐γ, TNF‐α, IL2, and Granzyme B were measured by flow after 1 day of recovery and 7 h of co‐culture. Data are representative of six independent experiments. CAR, chimeric antigen receptor; CD, cluster of differentiation; EP, electroporation; FSC, forward scatter; IFN, interferon; IL, interleukin; MFI, mean fluorescence intensity; TNF, tumor necrosis factor.  ^∗^ *p*  < 0.05,  ^∗∗^ *p*  < 0.01,  ^∗∗∗^ *p*  < 0.001,  ^∗∗∗∗^ *p*  < 0.0001 by *t*‐test (B, C1, C2, D1, D3, E) and ANOVA (A, C3, D2, D4). ns, nonsignificant.

The currently FDA‐approved CAR‐T production deploys cellular cryopreservation to improve the logistics of harvesting human cells and production. To assess the effect of cryopreservation/thawing on the cell viability and potency, we electroporated activated fresh human PBMCs (fresh EP) or cryopreserved them for 2 weeks before (cryopreserve + EP) and after (EP + cryopreserve) transfection. Using the three mRNA constructs, we continuously monitored cell viability for 72 h post‐EP or post‐thaw. We observed that the viability of the T cells transfected with CAR mRNA may decrease in the first 48 h for the fresh or cryopreserve + EP group; they recovered to a comparable level and exceeded 90% at 72 h. Interestingly, the EP + cryopreserve production yield steadily recovering cellular viability (Figure 5A). To assess the impact of cell cryopreservation on the receptivity of T cells to mRNA CAR EP and to determine whether CAR expression is affected in T cells that have undergone cryopreservation and recovery post‐EP, we performed the same EP procedure in both cryopreserve + EP and EP + cryopreserve groups. In the three experimental groups, with or without cell cryopreservation, there was no significant difference in the CAR expression levels (Figure 5B).

To study the impact of cellular cryopreservation on the tumor killing function of the mRNA‐based CAR‐T cells and its temporal stage of mRNA transfection, we compared the cryopreserve+EP and EP + cryopreserve groups. The results showed that the cryopreservation process did not have a significant impact on the mRNA‐based CAR‐T potency (Figures 5C and S5A–F). Cryopreserve + EP groups exhibited a four‐ to six‐fold of rapid T cell expansion (*p* < 0.0001, *p* < 0.0001, and *p* < 0.05 for three different CAR constructs respectively) similar to fresh EP group, however, the T cell expansion for EP + cryopreserve was reduced by nearly 50% (Figure 5D). Given the difference in T‐cell proliferation rates, we conducted T‐cell marker and cytokine production assays for the EP + cryopreserve group similar to the fresh EP group. Flow cytometry results indicate that for electroporated and cryopreserved CAR‐T cells, co‐culture with NALM6 similarly promotes the expression levels of CD69, Ki67, and various cytokines. Furthermore, the relative expression levels (compared to their respective electroporated and cryopreserved MOCK controls) are comparable to, or even slightly higher than, those in the fresh group. (Figure 5E). This suggests that the functional capacity of CAR‐T cells from the EP + cryopreserve group, as indicated by their activation upon target cell stimulation and cytokine secretion, remained unchanged. However, their proliferation rate was somewhat affected, possibly due to the need for some recovery time after cell thawing to resume normal division.

**Figure 6:**
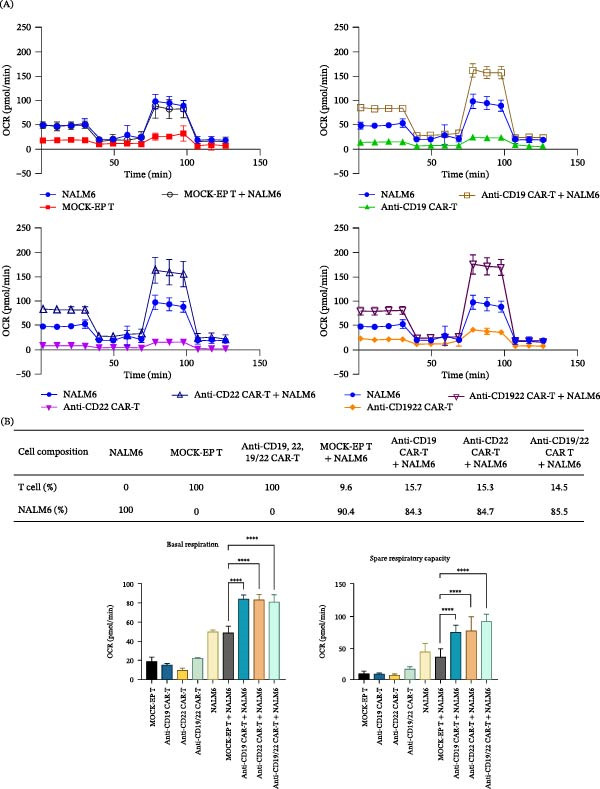
NALM6 stimulation significantly activates the cellular metabolic levels of CAR‐expressing T cells. Nonactivated human T cells from Donor 1 were electroporated with mRNA CAR and then co‐cultured with NALM6 at an E:T ratio of 1:1 for 4 days. Cell composition were detected by flow the same day when performing Seahorse assay. Seahorse using Cell Mito Stress Kit was performed (A) and basal respiration and spare respiratory capacity (B) were measured. CAR, chimeric antigen receptor; CD, cluster of differentiation; EP, electroporation; OCR, oxygen consumption rate.  ^∗^ *p*  < 0.05,  ^∗∗^ *p*  < 0.01,  ^∗∗∗^ *p*  < 0.001,  ^∗∗∗∗^ *p*  < 0.0001 by *t*‐test (B). ns, nonsignificant.

To assess the metabolism effect in the presence of the mRNA‐based CAR protein expression and activation, the nonactivated electroporated human T cells were co‐cultured with NALM6 cells at a 1:1 E:T ratio in the same medium for 4 days. In the presence of oligomycin, FCCP, and rotenone/antimycin A, mRNA‐based CAR‐T cells have at least 60% higher basal respiratory rate and two‐fold spare respiratory capacity (SRC) compared to the mock‐EP T cells in the presence of the target cells (Figure 6A,B). To further normalize these metabolic activities, modified oxygen consumption rate (OCR) was calculated. For modified basal respiration, MOCK‐EP T cells showed no significant difference with or without NALM6 stimulation, while all three types of CAR‐T cells exhibited a significant increase in basal respiration (18.1, 28.8 and 11.9 fold, respectively) after NALM6 stimulation (Figure S6A). Similarly, for SRC, NALM6 stimulation had no significant effect on the SRC of MOCK‐EP T cells, but it significantly increased the levels of CAR‐expressing T cells (*p* < 0.0001). Moreover, the SRC of the dual‐targeting anti‐CD19/22 CAR‐T cells was 55.9% and 44.4% higher than that of the single‐targeting anti‐CD19 (*p* < 0.001) and anti‐CD22 (*p* < 0.05) CAR‐T cells (Figure S6B).

## 3. Discussion

To our knowledge, this study provides the first extensive preclinical characterization of mRNA‐based CAR‐T cells co‐targeting CD19 and CD22, generated via a current good manufacturing practice (cGMP)‐compatible EP platform. Our findings demonstrate a substantially shortened manufacturing timeline of 2–3 days with CAR expression efficiencies of 66%–85% and high cell viability. The resulting CAR‐T cells exhibited robust in vitro cytotoxicity, antigen‐specific cytokine production, and enhanced metabolic activity upon tumor stimulation, with dual‐targeting CD19/22 CAR‐T cells showing distinct metabolic advantages. We also validated that short‐term cryopreservation does not compromise CAR‐T cell functionality. Upon successful in vivo validation, these findings could provide the foundation for further development of this platform as a rapid, cost‐effective, and nongenotoxic approach to CAR‐T generation.

Despite the achievements of virus‐based CAR‐T cells in clinical settings, safety concerns remain a significant issue as some degree of genotoxicity is unavoidable due to the integration of exogenous genes into the genome. Previously, there have been reports that in patients receiving CD19 CAR‐T, excessive CAR expression led to changes in gene copy numbers and insertions into the BACH2 and FYN genes [27], and among 10 patients in the trial, two developed CAR T tumors, resulting in the death of one individual. Meanwhile, CAR‐T therapy is often accompanied by adverse effects such as CRS, neurotoxicity, and cytopenia, which may further worsen the prognosis. Severe CRS manifestations could lead to significant morbidity and mortality of patients [28, 29]. In December 2023, the FDA issued a warning regarding virus‐based BCMA‐directed or CD19‐directed autologous CAR‐T immunotherapies [30]. All currently approved therapies carry the risk of CAR‐positive lymphoma, which has once again raised concerns and vigilance regarding the safety of virus‐based CAR‐T therapies. Thus, these known safety concerns have propelled the field to explore nonviral approaches in CAR‐T manufacturing.

As of now, while the application of mRNA‐based CAR T‐targeting CD19 in preclinical and clinical settings remains limited, the use of mRNA EP to introduce CAR genes has been widely utilized in the research field. A study [31] utilized in vitro transcribed (IVT) mRNA CAR with a single‐chain variable fragment (scFv) targeting CD19 (clone FMC‐63) or human mesothelin protein. The mRNA encoding CAR was introduced using the BTX CM380 electroporator (Harvard Apparatus BTX) at a concentration of 1 μg RNA per 10^6^ cells, reaching an efficiency of over 90%. The focus of this study was to compare the effects of the 4‐1BB and CD28 costimulatory domains within the same CAR structure on T cell proliferation and survival rates, T cell subset differentiation, and metabolic capacity. It did not directly correlate the mRNA EP technology with the killing ability of CAR‐T cells against tumors, cytokine secretion, or other functional aspects.

Additionally, Parayath et al. [32] have shown that they combine IVT mRNA CAR with nanocarrier delivery technology. Through these injectable nanocarriers, mRNA CAR can be delivered to circulating T cells in vivo, enabling transient expression of CARs capable of recognizing specific tumor antigens. This technology has shown promising results in mouse models targeting human leukemia, prostate cancer, hepatocellular tumors, and other disease models. While its safety profile and delivery specificity remain to be further evaluated, its application in CD19/CD22‐positive hematologic malignancies has yet to be fully explored. Currently, there is also a lack of a comprehensive systematic representation for mRNA‐based CAR‐T cells in B cell malignancies, spanning from manufacturing processes to cytotoxic functions, metabolic characteristics, and clinical application adaptability.

In our optimized protocol for generating mRNA‐based CAR‐T targeting CD19 positive malignancies via EP, we supplemented the culture medium with IL‐15 during the in vitro cultivation process, as studies have indicated that the membrane‐bound version of IL‐15 (mbIL15) can significantly enhance the persistence and anti‐tumor efficacy of CAR‐T cells in murine models [33], while a nonviral CAR‐T approach utilizing the PB transposon system as a vector showed an increase in expression efficiency from 20% to 40% after the addition of IL‐15 [34]. There is also evidence suggesting that a combination of cytokines, such as IL‐4, IL‐7, and IL‐21, can induce an immunophenotype in CAR‐T cells that tends toward an immature population. This approach may reduce the expression of exhaustion markers such as PD‐1 and TIM‐3 [35]. The CAR‐encoding IVT mRNA we utilized possesses a 5′ end cap structure using the m7G(5′)pppG cap analog [36] and a 3′ end poly A tail [37], enhancing stability and expression efficiency throughout the entire mRNA processing. The efficiency of EP is influenced by various factors, including voltage intensity, pulse frequency, pulse width, cell type, and activation status. In this study, we utilized the Maxcyte Gtx platform, which has been optimized for RNA delivery into primary human T cells and is compatible with GMP requirements for translational research. Recently, many studies have successfully applied the Maxcyte EP system in various fields, including using sgRNAs to gene edit CD34^+^ HSPCs [38] or producing CAR‐T^KO^ cells combining CRISPR and the Sleeping Beauty transposon systems [39], validating its feasibility and efficiency in RNA gene delivery operations.

As discussed earlier, the major limitations of CAR‐T cell manufacture based on viral transduction include high costs, a prolonged production cycle of 2–3 weeks, and a restricted production scale. In contrast, our mRNA‐based CAR‐T manufacture process, starting from the isolation of PBMCs to obtaining CAR‐T cells with functional CAR protein expression, takes only 2–3 days in total (Figure 1). This demonstrates a substantially shortened manufacturing timeline compared to that of conventional viral methods. Despite a recent study reporting that using lentivirus to directly transduce nonactivated T cells can also reduce the transduction process to 24 h, it still faces the issue that during CAR‐T cell harvest, the internal vector particles are still undergoing slow reverse transcription and integration, leaving the transduction incomplete [40]. In contrast, mRNA CAR‐T cells complete the entire transfection and expression process within a short period. Moreover, the manufacturing process does not involve virus culture, transduction, screening, et cetera. It only involves mRNA‐related procedures, eliminating the need for additional resources in facility and personnel training, thus significantly reducing the costs. Our protocol may also offer advantages in terms of scalability and cost reduction, although further validation in larger‐scale settings is needed. IVT mRNA, using plasmids containing the CAR gene sequence as a template, can be efficiently produced on a large scale. Many studies using lentivirus for transduction of a larger vector size have encountered limitations in transduction efficiency. We have validated that the expression level of mRNA may be positively correlated with the mRNA load within a certain range, and longer cargo sizes can enhance expression by increasing the mRNA load (Figures S1B and 2A). This suggests that multiple mRNA constructs or high cargo carrying capacity can be utilized, although the high limit of the threshold still needs to be determined. On the other hand, the plasmids serving as source materials can be rapidly amplified in bacteria, reducing costs by 5–10 times compared to viral processes [41].

The CAR‐T cells manufactured through mRNA EP also represent a transduced entity with transient expression. Their ability to bind to targets reaches its peak approximately 2 days post‐EP, followed by a rapid decline until the loss of CAR expression. This shortened duration of efficacy may also help avoid some side effects associated with the sustained expression of CAR genes in viral‐based long‐term CAR‐T cells, such as oncogenesis, CRS, and neurotoxicity in the patient’s body [17, 28]. Although transient expression is a crucial safety feature of mRNA‐based CAR‐T cells, it is generally believed that a significant drawback of mRNA‐based CAR‐T cells also lies in their transient profile. The rapid proliferation of activated T cells leads to the quick loss of this expression, limiting the therapeutic window and potentially resulting in weaker cytotoxic effects against tumor cells compared to those of methods involving stable transfection. The expression of CAR genes, when introduced into T cells in the form of mRNA, can be maintained for 7 days in the absence of T cell proliferation [42], a duration that has previously raised doubts about its efficacy in combating tumors. With our developed protocol, the CAR‐T cells manufactured exhibit approximately 66%–85% CAR expression (Figure 2A), maintained for about 48–72 h post EP (Figure 2B), demonstrating robust anti‐CD19 tumor killing activity and cytokine secretion in vitro (Figure 3). Therefore, this approach may represent a step toward a more cost‐effective manufacturing platform; however, its therapeutic efficacy relative to the current CAR‐T therapies remains to be evaluated in vivo. Looking forward, if validated in vivo, the rapid manufacturing timeline of mRNA‐based CAR‐T cells could make them particularly suitable for use as a bridging therapy in patients with advanced disease, potentially allowing for rapid tumor burden reduction prior to definitive treatments such as stem cell transplantation. However, this application remains conceptual until supported by appropriate animal models and, ultimately, clinical trials.

In the current clinical applications of CAR‐T therapy, T cells are typically isolated from the patient’s body and undergo CAR‐T cell manufacturing in specialized facilities. The process involves activation, transduction, expansion, purification, and subsequent reinfusion into the patient. During transportation, the cells are often cryopreserved. Therefore, it is crucial to validate whether the cryopreservation process affects the activity and functionality of CAR‐T cells. In our study, we investigated CAR expression (Figure 5B), tumor‐suppression functionality (Figure 5C), and cytokine production ability (Figure 5E) in CAR‐T cells that were either cryopreserved before EP or cryopreserved after EP. The results demonstrated no significant differences in functionality compared to freshly prepared CAR‐T cells. This implies that short‐term cryopreservation does not adversely impact the ability of our CAR‐T cells to exert therapeutic effects. This finding supports the feasibility of incorporating cryopreservation steps into future manufacturing workflows, should the approach advance to clinical testing.

The metabolic levels of CAR‐T cells are often closely related to their T cell function, exhaustion status, and other factors that significantly impact their antitumor performance. Until now, an increasing number of research teams have also been focusing on the influence of CAR expression on the metabolic profile of T cells. A number of studies explores different CAR structures, such as the CD28 and 4‐1BB co‐stimulatory domains [31], on the respiratory capacity of CAR‐T cells. There are also studies that optimize the metabolic profile of CAR‐T cells from different aspects by modulating gene expression in T cells or adjusting T cell subsets, such as T_SCM_ enrichment [43]. Research has reported that tonic‐signaling CAR‐expressing regulatory T cells (Tregs) exhibiting an exhaustion profile maintain a stable tumor‐suppressive effect in vitro, but they become nonfunctional in vivo [44]. The parameters commonly used for metabolic characterization include the SRC measured in Seahorse analysis. In our study, we also utilized Seahorse analysis to determine the metabolic differences exhibited by these mRNA‐based CAR‐T cells after stimulation with NALM6. While previous metabolic characterizations of CAR‐T cells have largely focused on lentivirally transduced products or compared different costimulatory domains, systematic metabolic profiling of mRNA‐electroporated CAR‐T cells—especially direct comparisons between single‐target CD19, single‐target CD22, and dual‐target CD19/CD22 configurations—has not been reported. To eliminate the influence of T cell activators on the basal metabolic levels of T cells, we used nonactivated T cells for EP and Seahorse experiments. Therefore, all the differences in metabolic levels observed are solely attributed to the CAR effect itself. All groups expressing CAR showed a significant increase in basal respiration and SRC after stimulation with NALM6, indicating a considerable degree of T cell activation by CAR recognition (Figure 6A). However, this activation phenomenon is relatively limited in MOCK‐EP T cells that do not express the CAR. We also combined the ratio of T cells to NALM6 in cell culture and used mathematical methods to normalize the data to obtain the theoretical expected values of basal respiration and SRC (SRC) for MOCK‐EP T cells and the three types of CAR‐T cells after NALM6 stimulation (Figure S6A,B). After such processing, the increased metabolic levels of CAR‐T cells in response to tumor stimulation were more pronounced; this represents the first direct comparison of mitochondrial respiratory capacity across CD19, CD22, and CD19/CD22 dual‐targeting mRNA CAR‐T cells. Additionally, we observed differential trends in SRC among the three CAR cell types, with the dual‐target CD19/22 CAR exhibiting higher SRC compared to the single‐target CARs, indicating greater metabolic potency. This observation raises the possibility that dual‐target CAR‐T cells may retain metabolic fitness under conditions of antigen escape, a hypothesis that warrants further investigation in vivo.

In the meantime, several other nonviral approaches are currently used to manufacture CAR‐T cells, employing various gene carriers, such as the Sleeping Beauty and piggyBac transposon systems, as well as nanocarriers, offering the capability to target and deliver exogenous genetic material to T cells in vivo, facilitating in vivo CAR‐T production. The introduction of exogenous genetic material into cells is primarily achieved also through EP. The evidence demonstrates that nonviral CAR‐T manufacturing platforms have achieved quantitative performance metrics approaching or matching viral vectors across multiple parameters. Key advantages include 4.5‐ to 9.3‐fold enhanced production yields with feeder support, comparable clinical complete remission rates (87.5%), and substantially reduced manufacturing costs and timelines [26, 45, 46]. While viral transduction maintains advantages in consistency and regulatory precedent, optimized nonviral approaches using transposon systems, advanced EP, or nanocarrier delivery have demonstrated transfection efficiencies of 43%–68% with >90% viability, therapeutic efficacy equivalent to viral vectors in preclinical models, and favorable safety profiles with low vector copy numbers [47–50]. The manufacturing method significantly influences the T cell phenotype, with implications for persistence, cytokine profiles, and exhaustion markers that warrant consideration in platform selection [51, 52]. However, the transposon system shares a defect similar to viral transduction, wherein the transposase enzyme is introduced into host cells in a trans‐configuration. This could potentially lead to the reactivation and mobilization of the transposon, resulting in the insertion of other gene sequences and compromising the genome integrity after transduction, posing concerning safety issues. In contrast, the approach utilizing mRNA transfection completely avoids integration with the genome. The CAR‐encoding construct is directly translated in the cytoplasm, allowing for the production of the CAR protein without the need for genomic integration. This fundamentally mitigates the safety concerns associated with the issues mentioned earlier. Studies indicate that using EP to introduce transposon systems with DNA as a carrier into T cells can result in lower transfection efficiency compared viral transduction, as one study reported a transduction efficiency of 30% [53, 54] and a need for 8‐day activation and cultivation required post‐EP [55], possibly due to the potential cytotoxicity of DNA during EP, which could decrease cell viability down to 40% 24 h post‐transfection. Our results demonstrate that after EP with mRNA CAR, cell viability was not significantly affected (Figure 5A), and expression efficiency maintained satisfactory. The two pilot clinical trials based on SB‐engineered CAR‐T cells currently in progress involve a prolonged in vitro cultivation period of approximately 28 days, requiring multiple stimulations with APCs and cytokines [56], while a long manufacturing process is believed to impair T cell function and efficacy. Our findings highlight the potential of mRNA EP to streamline CAR‐T manufacturing, which could contribute to reducing both time and cost barriers if future therapeutic development is feasible.

While the in vitro data presented here demonstrate the functional potency and manufacturing advantages of mRNA‐based CAR‐T cells, we acknowledge that the translational relevance of these findings remains to be established. Future investigations using xenograft mouse models of CD19/CD22‐positive malignancies will be essential to evaluate the antitumor efficacy, persistence, and safety profile of these mRNA‐based CAR‐T cells in a physiological context. Such studies will also help determine whether the metabolic advantages observed in dual‐target CAR‐T cells in vitro translate into improved tumor control in vivo. Only after rigorous preclinical evaluation in appropriate animal models can the potential clinical applicability of this approach be adequately assessed.

Looking forward, several key directions warrant further investigation to advance this mRNA‐based CAR‐T platform toward potential clinical applications. First and foremost, in vivo validation using xenograft mouse models of CD19/CD22‐positive malignancies would be instrumental to assess the antitumor efficacy, persistence, and safety in a physiological context. Second, direct comparative studies with established viral CAR‐T products and other nonviral platforms (e.g., Sleeping Beauty transposon and nanoparticle‐based delivery) under matched experimental conditions will be critical to rigorously benchmark the relative advantages and limitations of the mRNA approach, particularly regarding the trade‐off between safety and therapeutic durability. Third, optimization of targeting strategies, including combinations of tumor‐associated antigens, logic‐gated circuits, or integration with immune checkpoint modulation, may further enhance specificity and reduce the risk of relapse. Finally, from a clinical implication perspective, if successfully validated in vivo, this platform could be particularly suited for applications where rapid turnaround and enhanced safety are prioritized over long‐term persistence, such as bridging therapy to stem cell transplantation in patients with advanced or rapidly progressive disease or as a repeat‐dosing regimen to manage minimal residual disease. Continued refinement of manufacturing protocols, including scale‐up and automation, will also be necessary to realize the promise of a cost‐effective, accessible CAR‐T therapy for broader patient populations.

In summary, we have demonstrated that mRNA CAR‐T cells generated using EP can efficiently produce CAR‐T cells targeting anti‐CD19 and CD22 with an expression efficiency of 66%–85% within a short production cycle of 2–3 days. These CAR‐T cells exhibit robust tumor‐suppression capabilities and T cell functions, including cytokine production and cell respiration in vitro. While the mRNA‐based CAR‐T approach described here is unlikely to be curative as a monotherapy due to its transient expression profile, it could, if validated in vivo, find utility as a bridging strategy to enable subsequent stem cell transplantation—a concept that has been proposed in a previous study [57]. Moreover, short‐term cell cryopreservation before and after EP does not significantly impact the anti‐tumor functionality of CAR‐T cells. Collectively, these preclinical findings suggest that mRNA‐based CAR‐T cells warrant further investigation as a potentially rapid and low‐cost platform for CAR‐T generation. Future studies incorporating in vivo efficacy and toxicity models will be critical to assess whether these manufacturing advantages translate into meaningful clinical benefits.

## 4. Materials and Methods

### 4.1. mRNA Vector Encoding Anti‐CD19, Anti‐CD22, and Anti‐CD19/CD22 and *In Vitro* mRNA Transcription

CARs targeting human CD19, CD22, and dual‐targeting human CD19/22 on the 2^nd^ generation CAR backbone were employed. To generate in vitro‐transcribed RNA, the CAR‐encoding regions were subcloned onto plasmid vectors with T7 promoters‐pRh19v5c7, pRh2RF22C7, and pRh19h22C7, respectively (Figure S1C). Plasmid vectors were amplified in *E. coli*, extracted using a PureLink Quick Plasmid Miniprep Kit (Thermo Scientific), and linearized through SpeI/BcuI (Thermo Scientific) enzyme digestion. Subsequently, the linearized CAR‐encoding genes were transcribed into mRNA using the HiScribe T7 ARCA mRNA Kit (with tailing) (New England Biolabs).

### 4.2. Procurement and Manipulation of Healthy Human Donor Peripheral Blood

Consented healthy human donor peripheral blood was obtained under an approved institutional review board (IRB). Blood mononuclear cells were isolated using Ficoll‐Paque PLUS (GE Healthcare) and cultured in RPMI (Thermo Fisher Scientific) containing 50 ng/mL IL‐15 (Stem Cell Technology) and stimulated with ImmunoCult Human CD3/CD28/CD2 T Cell Activator (Stem Cell Technology, 25 μL/mL) for expansion and maintenance. Total viable cells and viability were measured by trypan blue staining.

### 4.3. EP of Human PBMCs

Using ExPERT GTx GMP Electroporator (Maxcyte), activated or nonactivated human PBMC or T lymphocytes were electroporated with mRNA CAR constructs at a concentration of 1 μg (anti‐CD19 and anti‐CD22 CAR) or 2 μg (anti‐CD19/22 CAR) per 10^6^ cells. Cells electroporated with no mRNA (MOCK‐EP) were used as a negative control. EP program expanded T cell 2 was used, as this technique was optimized for electroporating activated human T cells. Nonactivated PBMCs were electroporated using expanded T cell 4 via Maxcyte GTx. Electroporated T cells were rested in complete T cell media (RPMI supplemented with 10% FBS, 1% pen/strep, and 50 ng/mL IL‐15) for 18 h before further function analysis.

### 4.4. Immunophenotyping by Flow Cytometry

CAR expression was evaluated by staining with AF647‐Recombinant Human CD19 Protein and FITC‐Recombinant Human Siglec‐2/CD22 Fc Protein (R&D Systems), respectively. Cells were washed with PBS once after staining and resuspended in PBS. Flow cytometric analysis was performed using Cytoflex (Beckman Coulter) and data were analyzed with FlowJo v10.10.1 (Beckton, Dickinson and Company). The gating strategy was as follows: cellular debris and doublets were first excluded based on forward scatter (FSC) and side scatter (SSC) properties to gate on the target cell population. Viable cells were then selected by gating on 7‐aminoactinomycin D (7‐AAD)‐negative events. Within the viable cell population, NALM6 tumor cells and T cells were distinguished based on their GFP expression profiles, as NALM6 cells were engineered to express GFP and T cells were GFP‐negative. T cell subpopulation and activation markers were evaluated using anti‐CD4‐BV510 (Biolegend), anti‐CD8‐PE (Biolegend) and anti‐CD69‐BV605 (Biolegend). Intracellular cytokine production was measured after 7 h of co‐culture, Brefeldin A (1 μL per 10^6^ cells) was added to the cell culture 2 h after the initiation of co‐culture, followed by cell fixation and permeabilization with the Cytofix/CytopermTM Fixation/Permeabilization Kit (BD Biosciences). T cell activation‐induced intracellular cytokine and protein production was measured using IFN‐γ‐PE/cyanine 7 (Biolegend), TNF‐α‐PE/Dazzle 594 (Biolegend),IL‐2‐BV421 (Biolegend), and Granzyme B‐AF700 (Biolegend). Cell cycle marker, Ki67‐PE/Cy 5.5 (eBioscience), was used to evaluate cell proliferation (Figure S7).

### 4.5. NALM6 Killing Assay

CAR‐T cells or control T cells were mixed and co‐cultured with ZsGreen NALM6 cells at E:T ratios of 1:1, 1:3, 1:5, and 1:10, 24 h post‐EP in a 96‐well plate. The number of cells seeded in the experimental group is based on the actual count of cells expressing the CAR protein. Using Revolve2‐K1105 (ECHO), fluorescent images were captured in the FITC channel for each well where cells exhibiting green fluorescence correspond to NALM6 cell presence.

### 4.6. Cell Cryopreservation and Thawing

Fresh PBMCs to be cryopreserved were centrifuged at 1200 rpm for 10 min and resuspended in prechilled RPMI containing 20% FBS (Corning) and 10% DMSO (Corning). They were then aliquoted into cryovials, transferred to a −80 freezer overnight and then stored in liquid nitrogen until needed for thaw. For EP + cryopreserve group, T cells electroporated with mRNA CAR were incubated in RPMI media for recovery for 2 h before cryopreservation and centrifuged at a lower speed of 95 × *g* (650 rpm) before a similar cryopreservation procedure. For thawing the cells, the cryovials were rapidly thawed in 37°C water bath, diluted in RPMI complete media at 10 times the volume, centrifuged at 1200 rpm for 10 min, and then resuspended at a concentration of 5 × 10^5^–10^6^ per mL in pre‐warmed RPMI medium supplemented with IL‐15 and ImmunoCult Human CD3/CD28/CD2 T Cell Activator (Stem Cell Technology).

### 4.7. Cellular Metabolism Assay

Human PBMCs were co‐cultured with NALM6 at an E:T ratio of 1:1 directly for 4 days. On the same day as conducting the Seahorse analysis, flow cytometry was performed on each co‐culture group to determine the T cell and NALM6 composition. Seahorse using Cell Mito Stress Kit (Agilent) was performed on NALM6, MOCK‐EP T, anti‐CD19 CAR‐T, anti‐CD22 CAR‐T, anti‐CD19/22 CAR‐T, and four co‐cultured groups; 150k cells were seeded in each well, and basal respiration and SRC were measured. Modified basal respiration and SRC for T cells only were calculated by the formula: modified OCR = (OCR for mixed cells − OCR for NALM6 only × NALM6 percentage)/T cell percentage.

### 4.8. Statistical Analysis

All statistical analyses were performed using GraphPad Prism (GraphPad Software, San Diego, CA, USA). Data are presented as the mean ± SEM unless otherwise indicated. For comparisons between two groups, the unpaired two‐tailed Student’s *t*‐test was used for parametric data. For multiple group comparisons, one‐way or two‐way analysis of variance (ANOVA) was applied, followed by appropriate post hoc corrections, as specified in the respective figure legends. Sample sizes were determined based on preliminary pilot experiments to ensure adequate statistical power. A *p*‐value of less than 0.05 was considered statistically significant.

## Author Contributions

Chenyun Zhang, Haizhou Liu, and Hrithik Sangani have performed the experiments, data collection, and analysis. David Jin and Yen‐Michael S. Hsu have conceptualized, designed the manufacturing protocol, and the mRNA vectors used in the research. David Jin provided the mRNA vectors material to be used in the research. Chenyun Zhang and Yen‐Michael S. Hsu have developed the research hypothesis/aims, verified results, statistical analysis, and confirming findings.

## Funding

Yen‐Michael S. Hsu is supported by the Hillman Cancer Center Senior Fellowship for Innovative Cancer Research, Chenyun Zhang is supported by the Tsinghua University School of Medicine Collaborative Education and Research Agreement (Scholar Group 10, 2022–2024) through the University of Pittsburgh, and this work was supported by the sponsored research agreement (Avalon Globocare Corp.). The study utilized the UPMC Hillman Cancer Center Immunologic Monitoring and Cellular Products Laboratory, supported by the CCSG (Grant P30CA047904).

## Disclosure

The authors have reviewed and verified the content and take full responsibility for the final manuscript.

## Ethics Statement

Consented healthy human donor peripheral blood was obtained under an approved institutional review board (IRB) protocol at the University of Pittsburgh Medical Center Hillman Cancer Center.

## Consent

The authors have nothing to report.

## Conflicts of Interest

The authors declare no conflicts of interest.

## Supporting Information

Additional supporting information can be found online in the Supporting Information section.

## Supporting information


**Supporting Information** Supplemental Figures S1–S7 are available in the supporting file provided with this manuscript. Figure S1. (A) Human PBMCs were activated by immunocult human CD3/CD28/CD2 T cell activator and expression of CD4, CD8, and CD69 were detected via flow before and after 30 h of activation. Data are representative of three independent experiments. (B) Jurkat were electroporated with 1, 10, 100, and 500 ng/uL GFP mRNA and GFP fluorescence were detected by flow 24 h post‐EP. Structure of three CAR‐encoding plasmids using second generation CAR backbone with CD3 and 4‐1BB co‐stimulatory domain. CAR, chimeric antigen receptor; CD, cluster of differentiation; EP, electroporation; FSC, forward scatter; GFP, green fluorescent protein; scFv, single‐chain variable fragment.  ^∗^ *p* < 0.05,  ^∗∗^ *p* < 0.01,  ^∗∗∗^ *p* < 0.001,  ^∗∗∗∗^ *p* < 0.0001 by *t*‐test (A). Figure S2. T cells were cryopreserved for two weeks before (A) or after (B) EP. CAR expression were detected by AF647‐CD19 and FITC‐CD22 from 24 to 72 h post‐EP (A) or post‐thaw (B). Expression rate of CD19/22 CAR‐T includes the population of both CD19‐CAR and CD22‐CAR monopositive cells in flow cytometry. Data are representative of three independent experiments. CAR, chimeric antigen receptor; CD, cluster of differentiation; EP, electroporation.  ^∗^ *p* < 0.05,  ^∗∗^ *p* < 0.01,  ^∗∗∗^ *p* < 0.001,  ^∗∗∗∗^ *p* < 0.0001 by ANOVA (A, B). Figure S3. The cytotoxic effect of mRNA‐based CAR‐T cells is tumor‐specific. (A) mRNA CAR‐T cells were cultured in vitro for 96 h after EP and then co‐cultured with ZsGreen‐labeled NALM6 at an E:T ratio of 10:1 (250k + 25k), 5:1 (125k + 25k), 3:1 (75k + 25k), 1:1 (50k + 50k), 1:3 (25k + 75k), 1:5 (25k + 125k), and 1:10 (25k + 125k). NALM6 expansion fold (normalized to NALM6 only) were measured by flow after 24 h of co‐culture. Data are representative of two independent experiments. (B) mRNA CAR‐T cells were co‐cultured with MDA‐MB‐231 cells 24 h post‐EP at an E:T ratio of 1:1 for 24 h, MDA‐MB cell count were measured after 24 h of co‐culture. Data are representative of three independent experiments. CAR, chimeric antigen receptor; CD, cluster of differentiation; EP, electroporation.  ^∗^ *p* < 0.05,  ^∗^ *p* < 0.01,  ^∗∗∗^ *p* < 0.001,  ^∗∗∗∗^ *p* < 0.0001 by ANOVA (A, B). ns, nonsignificant. Figure S4. Anti‐CD19 CAR T cells generated from Donor 1 and NALM6 cells were co‐cultured at an E:T ratio of 1:1 24 h post‐EP, CD8^+^ cell count were measured by flow and cell counting after 0, 7, 24, and 48 h of co‐culture. Data are representative of three independent experiments. CAR, chimeric antigen receptor; CD, cluster of differentiation; EP, electroporation.  ^∗^ *p* < 0.05,  ^∗∗^ *p* < 0.01,  ^∗∗∗^ *p* < 0.001,  ^∗∗∗∗^ *p* < 0.0001 by *t*‐test. ns, nonsignificant. Figure S5. mRNA CAR‐T cells generated from donor 1 that have been cryopreserved before (A, C) and after (B, D) EP were co‐cultured with ZsGreen‐labeled NALM6 at an E:T ratio of 1:1 (50k + 50k), 1:3 (25k + 75k), 1:5 (25k + 125k), and 1:10 (25k + 125k). Effector cell count was normalized based on the actual CAR^+^ cells. Residual NALM6 (A, B) were measured by flow, and fluorescence pictures (C, D) were taken after 24 h of co‐culture. (E, F) For CAR T cells cryopreserved after EP, CD69 and Ki67 (E) and intracellular production of cytokines IFNγ, TNF‐α, IL2, and Granzyme B (F) were measured by flow after 1 day of recovery and 7 h of co‐culture. CAR, chimeric antigen receptor; CD, cluster of differentiation; EP, electroporation; FSC, forward scatter; GFP, green fluorescent protein; IFN, interferon; IL, interleukin; TNF, tumor necrosis factor. Figure S6. Modified basal respiration (A) and spare respiratory capacity (B) for T cell only was calculated by formula: modified OCR = (OCR for mixed cells − OCR for NALM6 only × NALM6 percentage)/T cell percentage. CAR, chimeric antigen receptor; CD, cluster of differentiation; EP, electroporation; OCR, oxygen consumption rate.  ^∗^ *p* < 0.05,  ^∗∗^ *p* < 0.01,  ^∗∗∗^ *p* < 0.001,  ^∗∗∗∗^ *p* < 0.0001 by *t*‐test (A, B). ns, nonsignificant. Figure S7. Cell population were gated based on FSC‐H and SSC‐H, single cell population were gated based on FSC‐H and FSC‐A. Live cells were then selected by gating on 7‐AAD‐negative events. Within the live cell population, NALM6 tumor cells and T cells were distinguished based on GFP expression. All T cell markers and cytokine production were analyzed under the T cell gate. 7‐AAD, 7‐aminoactinomycin D; FSC, forward scatter; SSC side scatter.

## Data Availability

All the data generated or analyzed during this study are included in this published article.
