# Supplementary material for: Characterization of Short Production Cycle and Nongenotoxic mRNA‐Based CAR‐T Cells Targeting CD19‐Positive and CD22‐Positive Malignancies
Source: J Immunol Res. 2026 Jul 30;2026:9452254. doi: 10.1155/jimr/9452254 (PMC13424616; doi:10.1155/jimr/9452254)
Supplement: Supplementary file 1 — Supporting Information Supplemental Figures S1–S7 are available in the supporting file provided with this manuscript. Figure S1. (A) Human PBMCs were activated by immunocult human CD3/CD28/CD2 T cell activator and expression of CD4, CD8, and CD69 were detected via flow before and after 30 h of activation. Data are representative of three independent experiments. (B) Jurkat were electroporated with 1, 10, 100, and 500 ng/uL GFP mRNA and GFP fluorescence were detected by flow 24 h post‐EP. Structure of three CAR‐encoding plasmids using second generation CAR backbone with CD3 and 4‐1BB co‐stimulatory domain. CAR, chimeric antigen receptor; CD, cluster of differentiation; EP, electroporation; FSC, forward scatter; GFP, green fluorescent protein; scFv, single‐chain variable fragment. ∗ p < 0.05, ∗∗ p < 0.01, ∗∗∗ p < 0.001, ∗∗∗∗ p < 0.0001 by t‐test (A). Figure S2. T cells were cryopreserved for two weeks before (A) or after (B) EP. CAR expression were detected by AF647‐CD19 and FITC‐CD22 from 24 to 72 h post‐EP (A) or post‐thaw (B). Expression rate of CD19/22 CAR‐T includes the population of both CD19‐CAR and CD22‐CAR monopositive cells in flow cytometry. Data are representative of three independent experiments. CAR, chimeric antigen receptor; CD, cluster of differentiation; EP, electroporation. ∗ p < 0.05, ∗∗ p < 0.01, ∗∗∗ p < 0.001, ∗∗∗∗ p < 0.0001 by ANOVA (A, B). Figure S3. The cytotoxic effect of mRNA‐based CAR‐T cells is tumor‐specific. (A) mRNA CAR‐T cells were cultured in vitro for 96 h after EP and then co‐cultured with ZsGreen‐labeled NALM6 at an E:T ratio of 10:1 (250k + 25k), 5:1 (125k + 25k), 3:1 (75k + 25k), 1:1 (50k + 50k), 1:3 (25k + 75k), 1:5 (25k + 125k), and 1:10 (25k + 125k). NALM6 expansion fold (normalized to NALM6 only) were measured by flow after 24 h of co‐culture. Data are representative of two independent experiments. (B) mRNA CAR‐T cells were co‐cultured with MDA‐MB‐231 cells 24 h post‐EP at an E:T ratio of 1:1 for 24 h, MDA‐MB cell count were [file JIMR-2026-9452254-s001.docx]

Supplemental


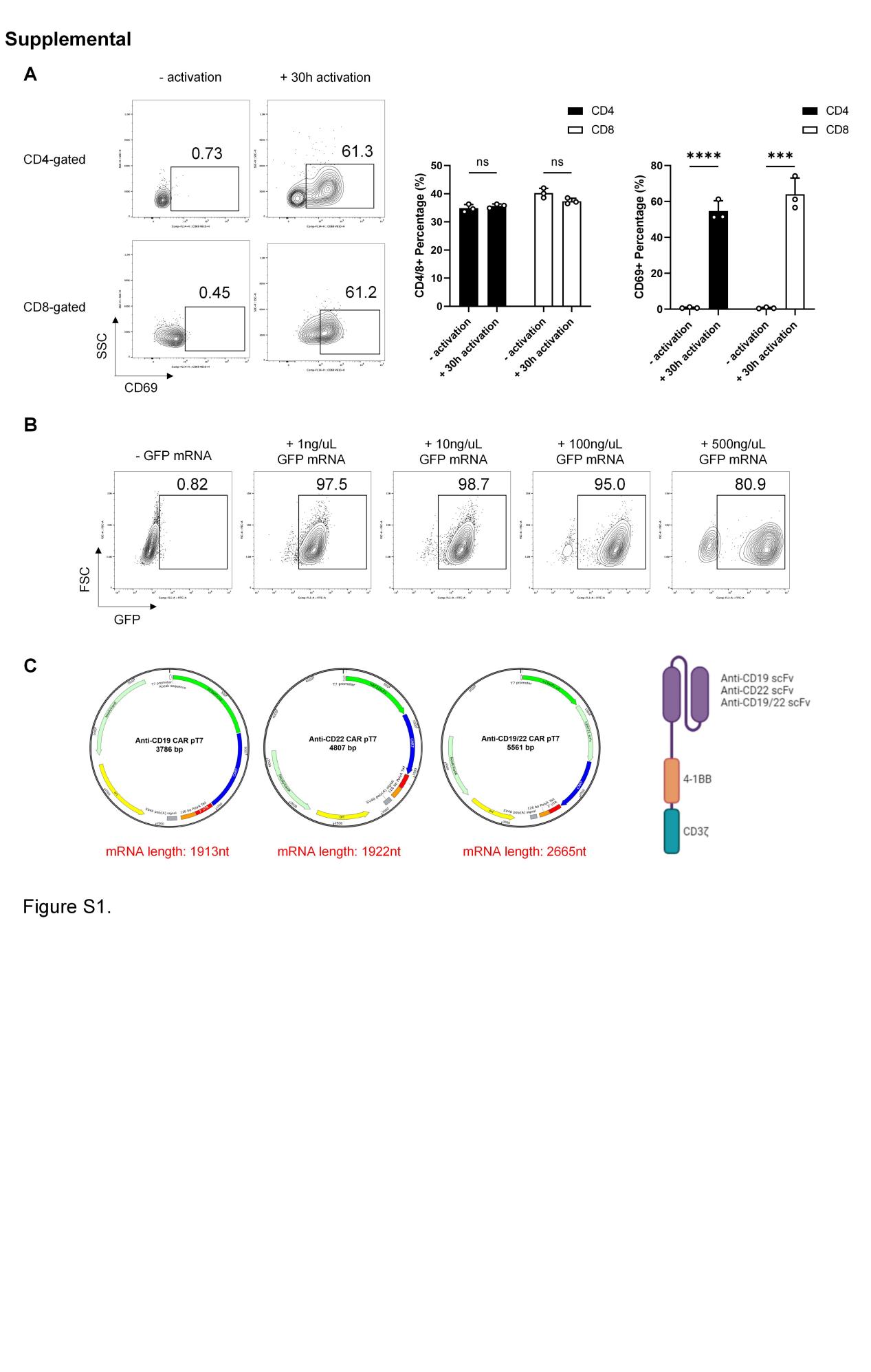


Figure S1.

(A) Human PBMCs were activated by Immunocult Human CD3/CD28/CD2 T Cell Activator and expression of CD4, CD8 and CD69 were detected via flow before and after 30h of activation. Data are representative of three independent experiments. (B) Jurkat were electroporated with 1ng/uL, 10ng/uL, 100ng/uL and 500 ng/uL GFP mRNA, and GFP fluorescence were detected by flow 24h post-EP. Structure of three CAR-encoding plasmids using second generation CAR backbone with CD3 and 4-1BB co-stimulatory domain. Abbreviations: CAR, chimeric antigen receptor; CD, cluster of differentiation; EP, electroporation; FSC, forward scatter; GFP, green fluorescent protein; scFv, single-chain variable fragment.

*p<0.05, **p<0.01, ***p<0.001, ****p<0.0001 by t-test (A).


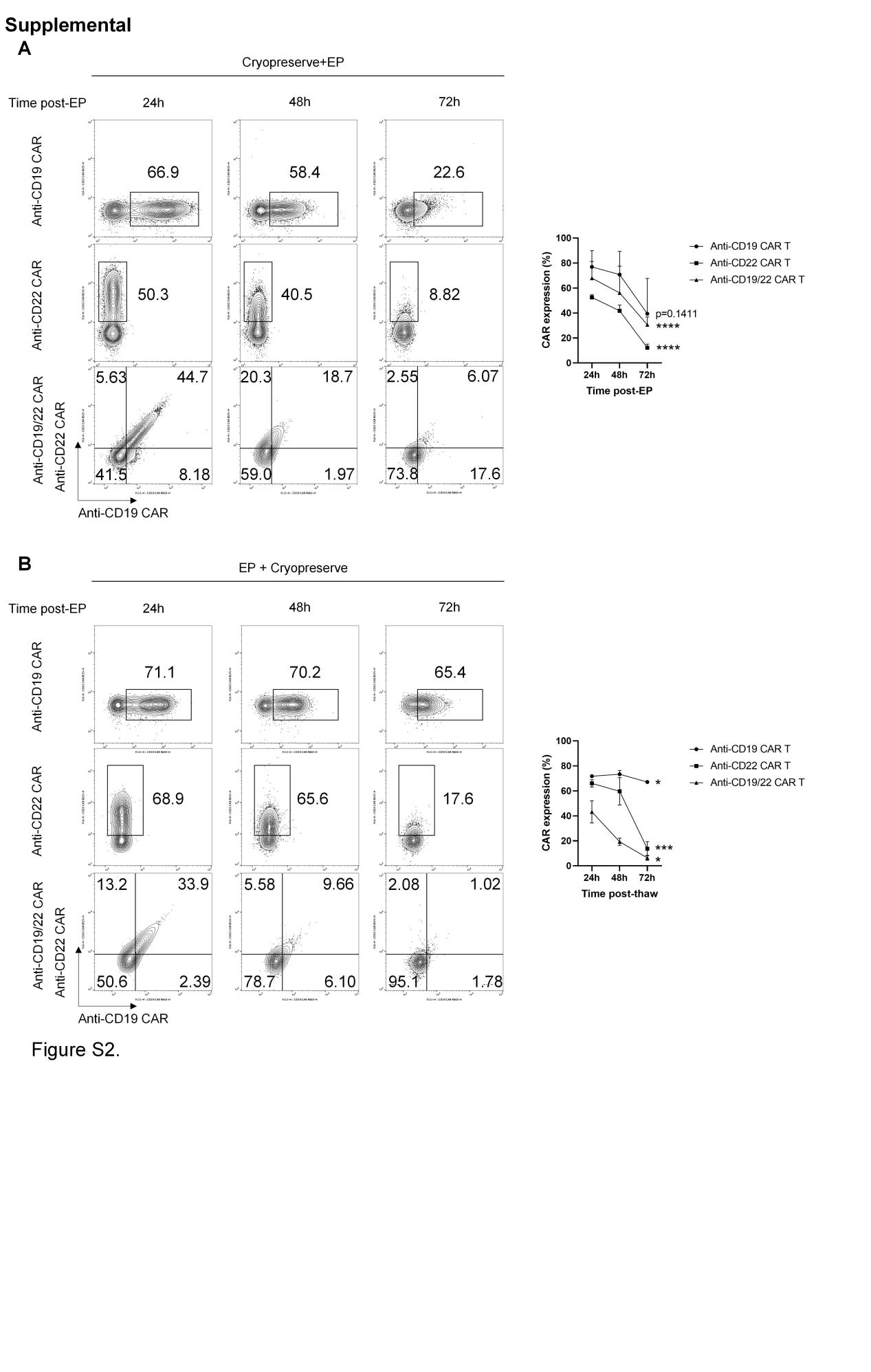


Figure S2.

T cells were cryopreserved for two weeks before (A) or after (B) EP. CAR expression were detected by AF647-CD19 and FITC-CD22 from 24h to 72h post-EP (A) or post-thaw (B). Expression rate of CD19/22 CAR-T includes the population of both CD19-CAR and CD22-CAR monopositive cells in flow cytometry. Data are representative of three independent experiments. Abbreviations: CAR, chimeric antigen receptor; CD, cluster of differentiation; EP, electroporation.

*p<0.05, **p<0.01, ***p<0.001, ****p<0.0001 by ANOVA (A, B).


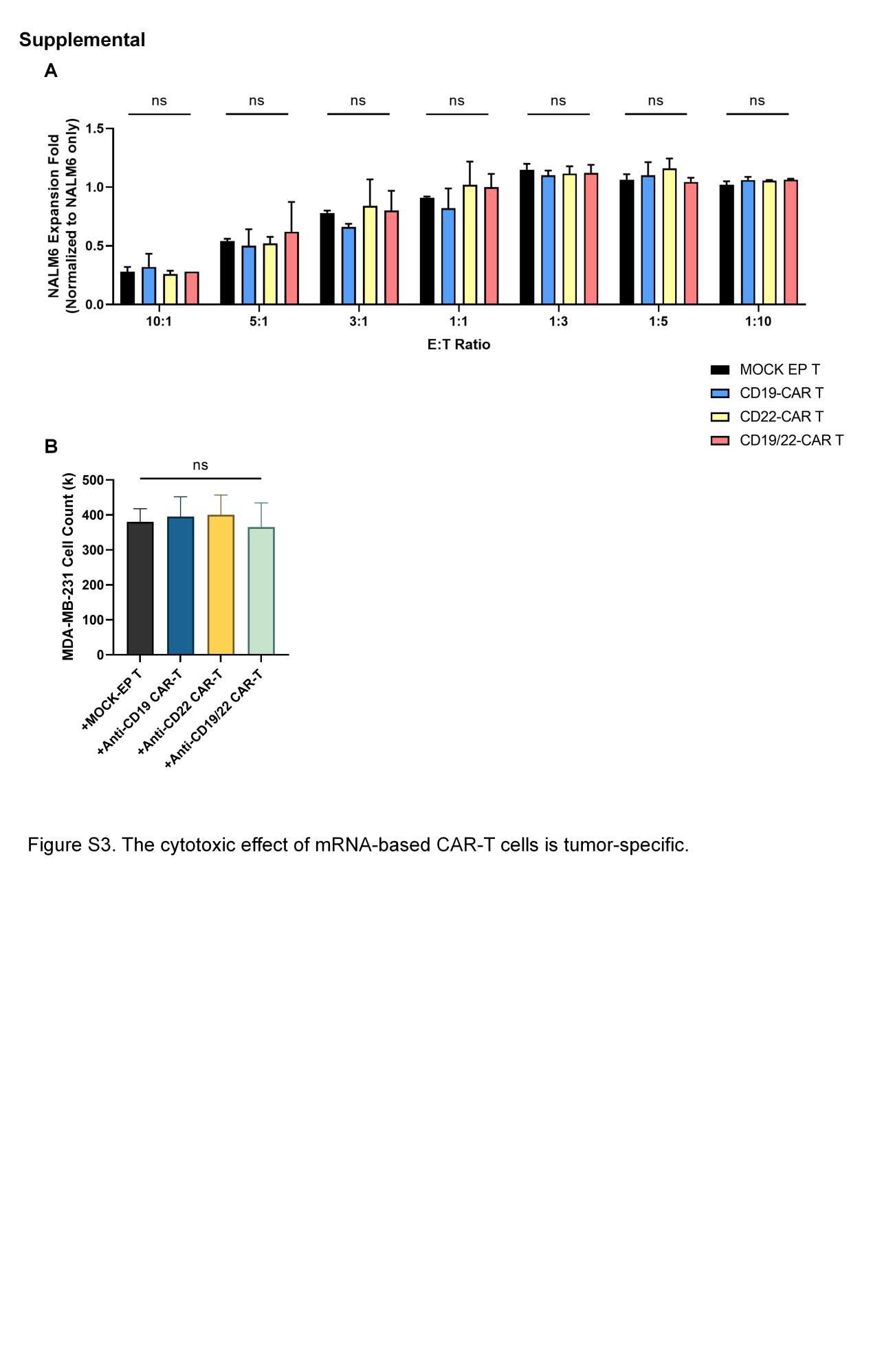


Figure S3. The cytotoxic effect of mRNA-based CAR-T cells is tumor-specific.

(A) mRNA CAR-T cells were cultured in vitro for 96h after EP and then co-cultured with ZsGreen-labeled NALM6 at an E:T ratio of 10:1 (250k+25k), 5:1 (125k+25k), 3:1 (75k+25k), 1:1 (50k+50k), 1:3 (25k+75k), 1:5 (25k+125k) and 1:10 (25k+125k). NALM6 expansion fold (normalized to NALM6 only) were measured by flow after 24h of co-culture. Data are representative of two independent experiments. (B) mRNA CAR-T cells were co-cultured with MDA-MB-231 cells 24h post-EP at an E:T ratio of 1:1 for 24h, MDA-MB cell count were measured after 24h of co-culture. Data are representative of three independent experiments. Abbreviations: CAR, chimeric antigen receptor; CD, cluster of differentiation; EP, electroporation.

*p<0.05, *p<0.01, ***p<0.001, ****p<0.0001 by ANOVA (A, B). ns, non-significant.


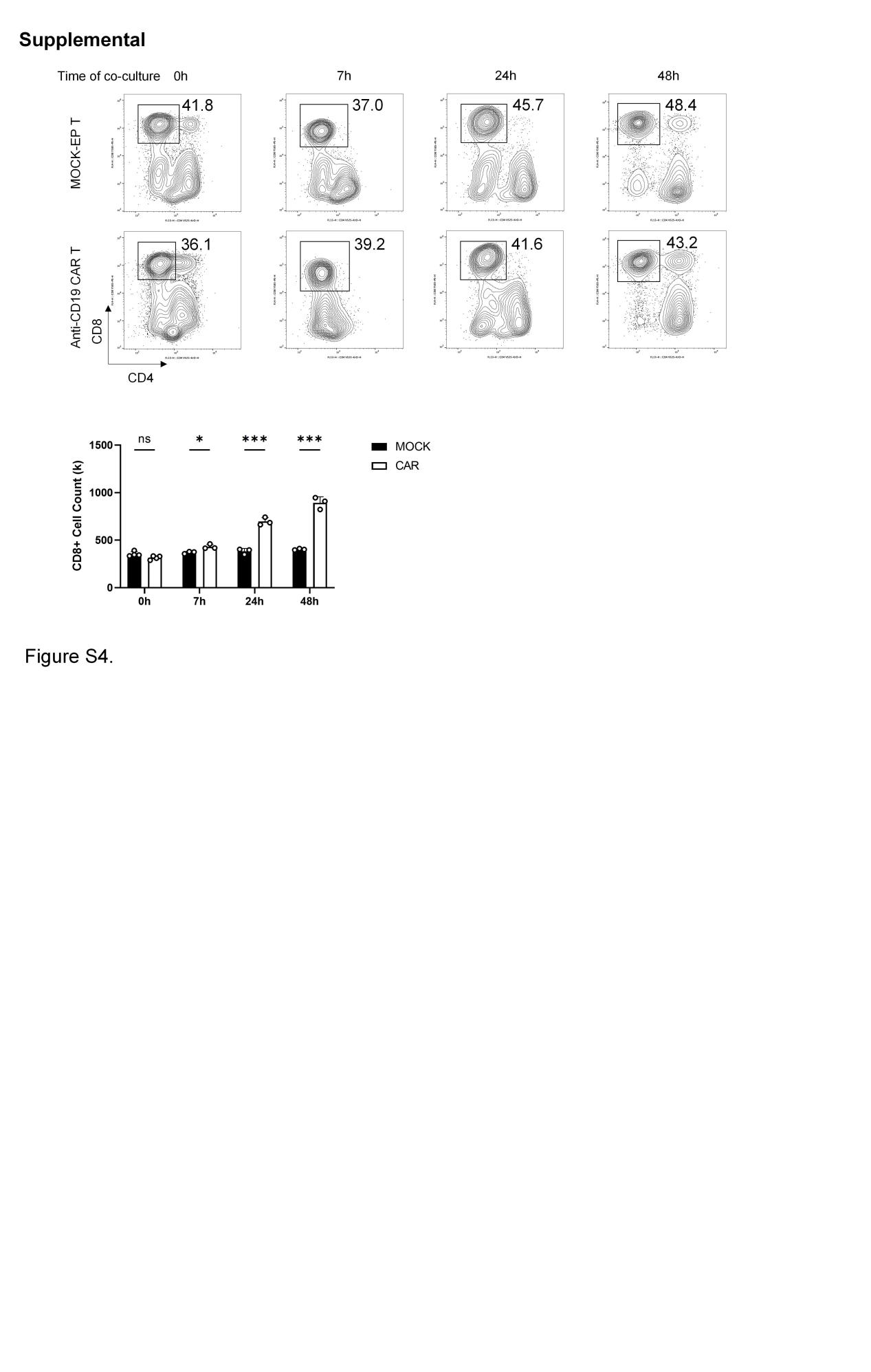


Figure S4.

Anti-CD19 CAR T cells generated from Donor 1 and NALM6 cells were co-cultured at an E:T ratio of 1:1 24h post-EP, CD8+ cell count were measured by flow and cell counting after 0h, 7h, 24h and 48h of co-culture. Data are representative of three independent experiments. Abbreviations: CAR, chimeric antigen receptor; CD, cluster of differentiation; EP, electroporation.

*p<0.05, **p<0.01, ***p<0.001, ****p<0.0001 by t-test. ns, non-significant.


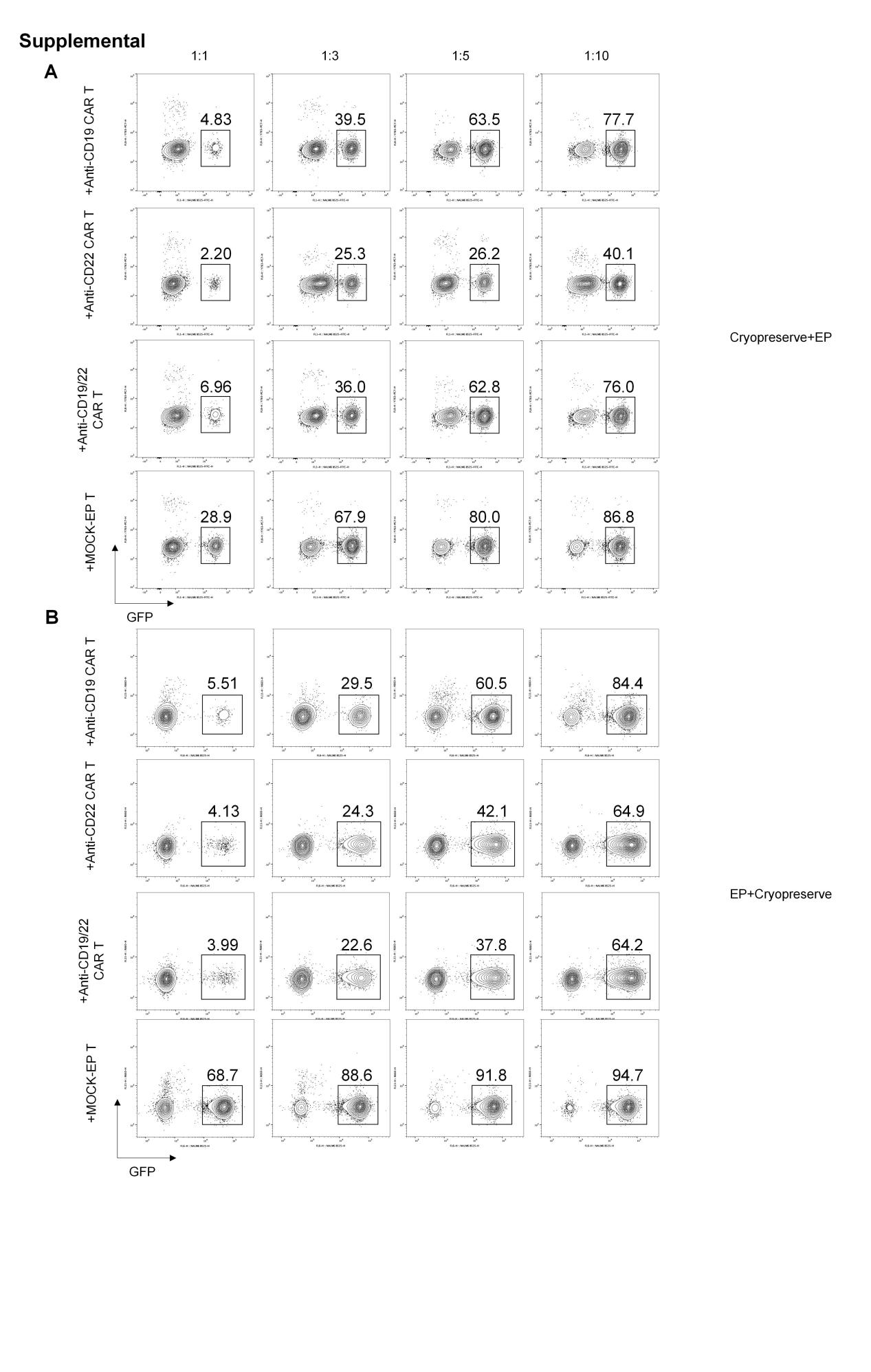


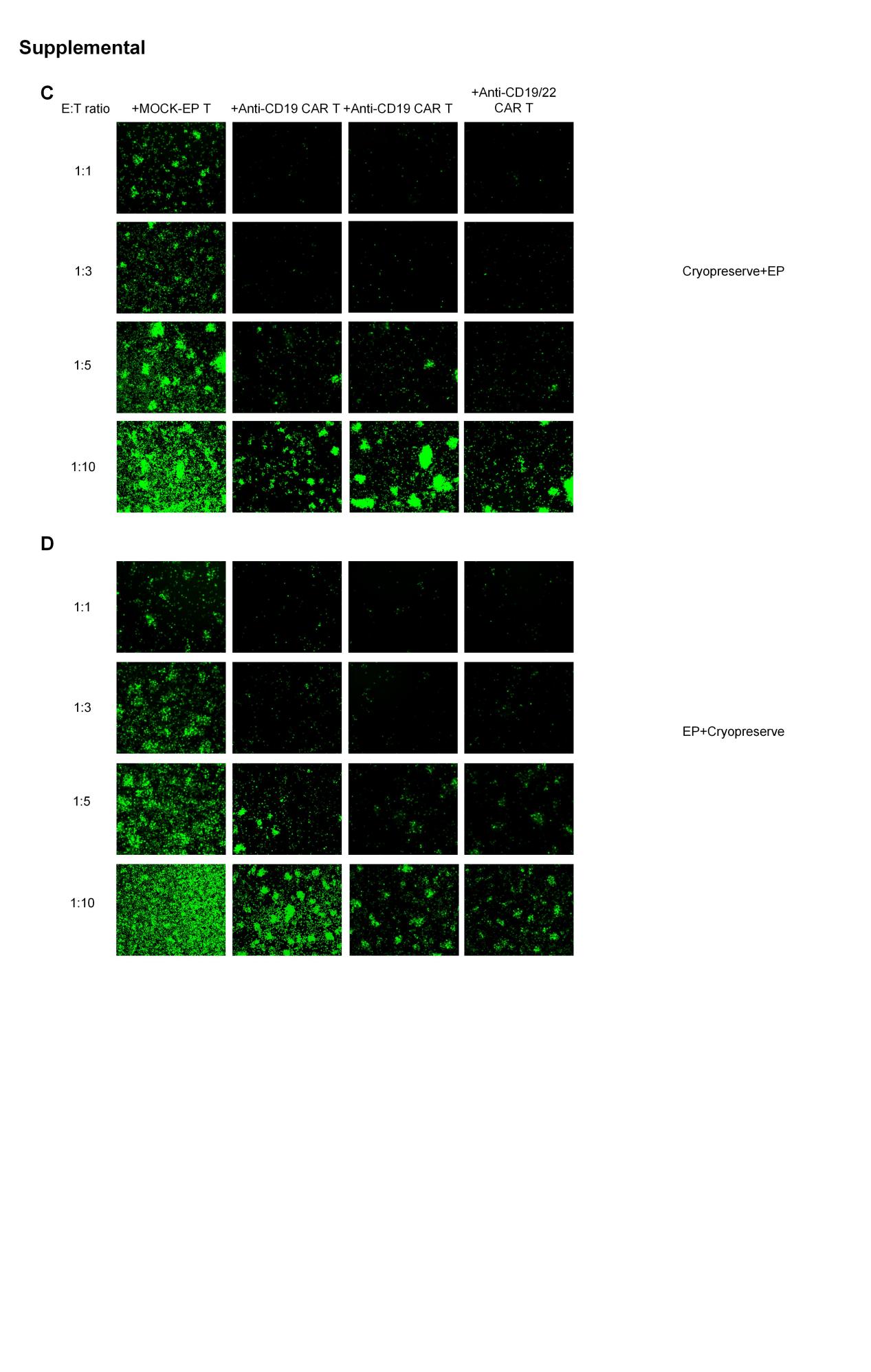


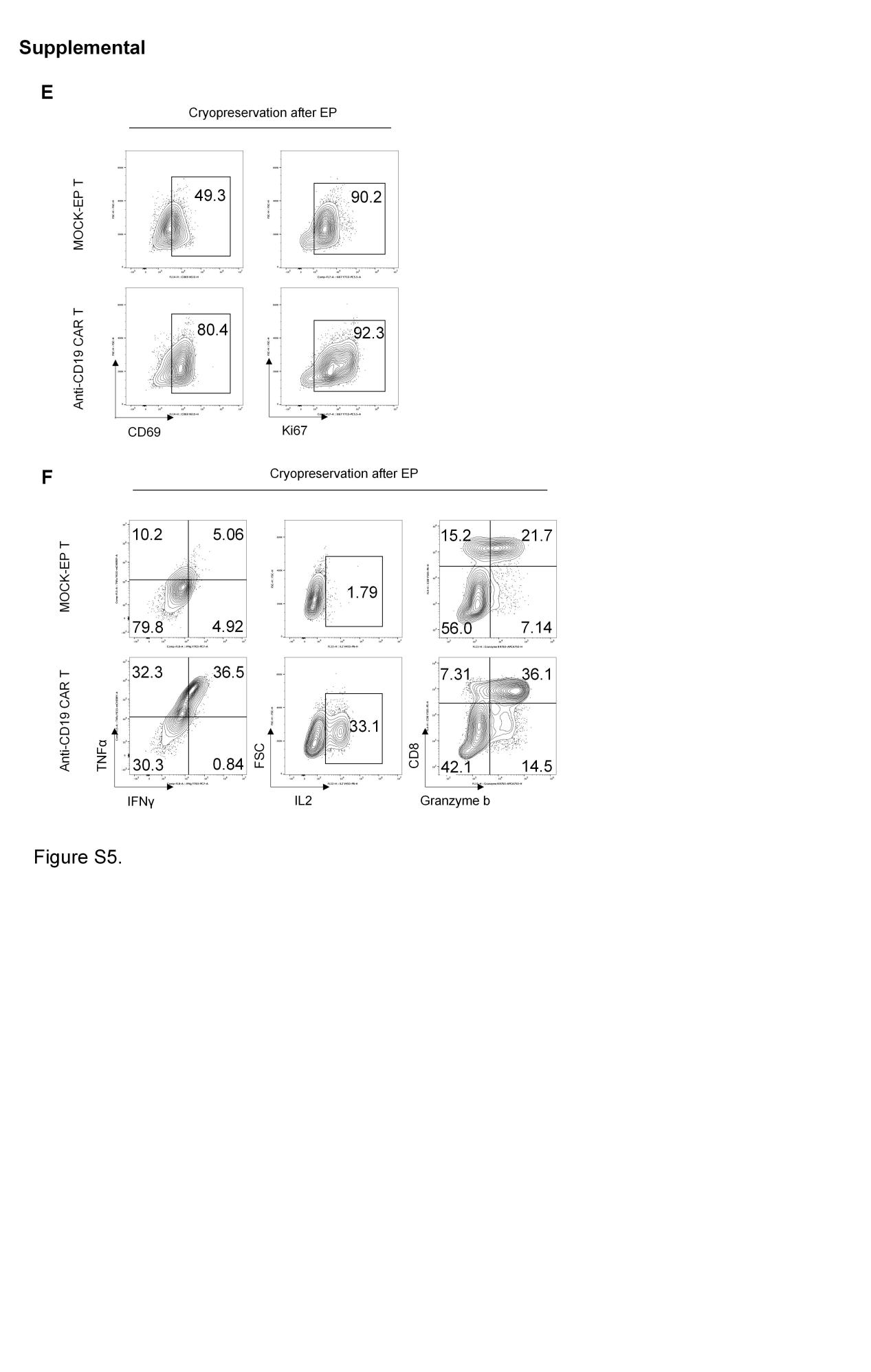


Figure S5.

mRNA CAR-T cells generated from donor 1 that have been cryopreserved before (A, C) and after (B, D) EP were co-cultured with ZsGreen-labeled NALM6 at an E:T ratio of 1:1 (50k+50k), 1:3 (25k+75k), 1:5 (25k+125k) and 1:10 (25k+125k). Effector cell count was normalized based on the actual CAR+ cells. Residual NALM6 (A, B) were measured by flow, and fluorescence pictures (C, D) were taken after 24h of co-culture. (E, F) For CAR T cells cryopreserved after EP, CD69 and Ki67 (E), and intracellular production of cytokines IFNγ, TNFα, IL2, Granzyme B (F) were measured by flow after 1 day of recovery and 7h of co-culture. Abbreviations: CAR, chimeric antigen receptor; CD, cluster of differentiation; EP, electroporation; FSC, forward scatter; GFP, green fluorescent protein; IFN, interferon; IL, interleukin; TNF, tumor necrosis factor.


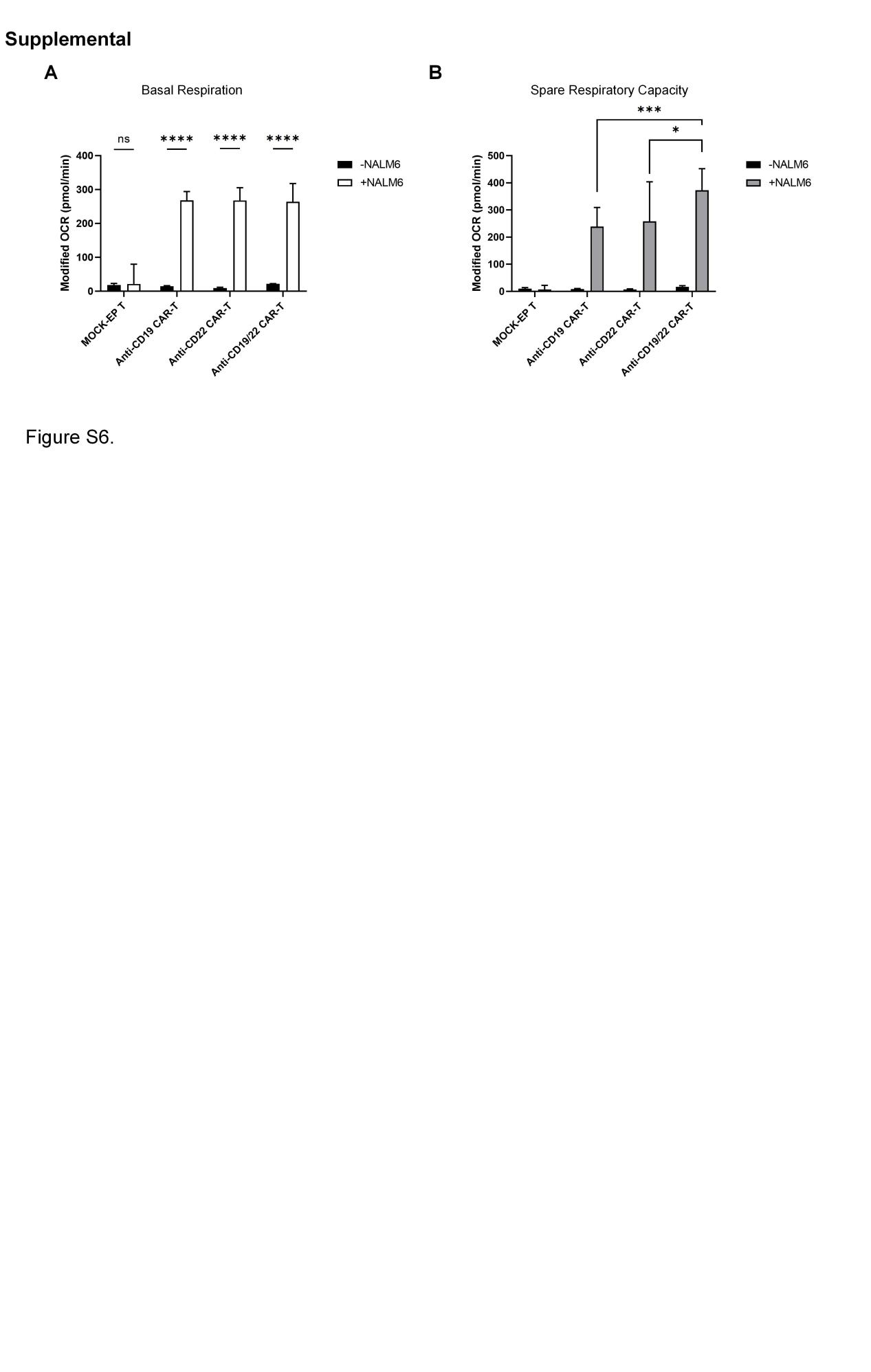


Figure S6.

Modified basal respiration (A) and spare respiratory capacity (B) for T cell only was calculated by formula: Modified OCR = (OCR for mixed cells - OCR for NALM6 only×NALM6 percentage)/T cell percentage. Abbreviations: CAR, chimeric antigen receptor; CD, cluster of differentiation; EP, electroporation; OCR, oxygen consumption rate.

*p<0.05, **p<0.01, ***p<0.001, ****p<0.0001 by t-test (A, B). ns, non-significant.


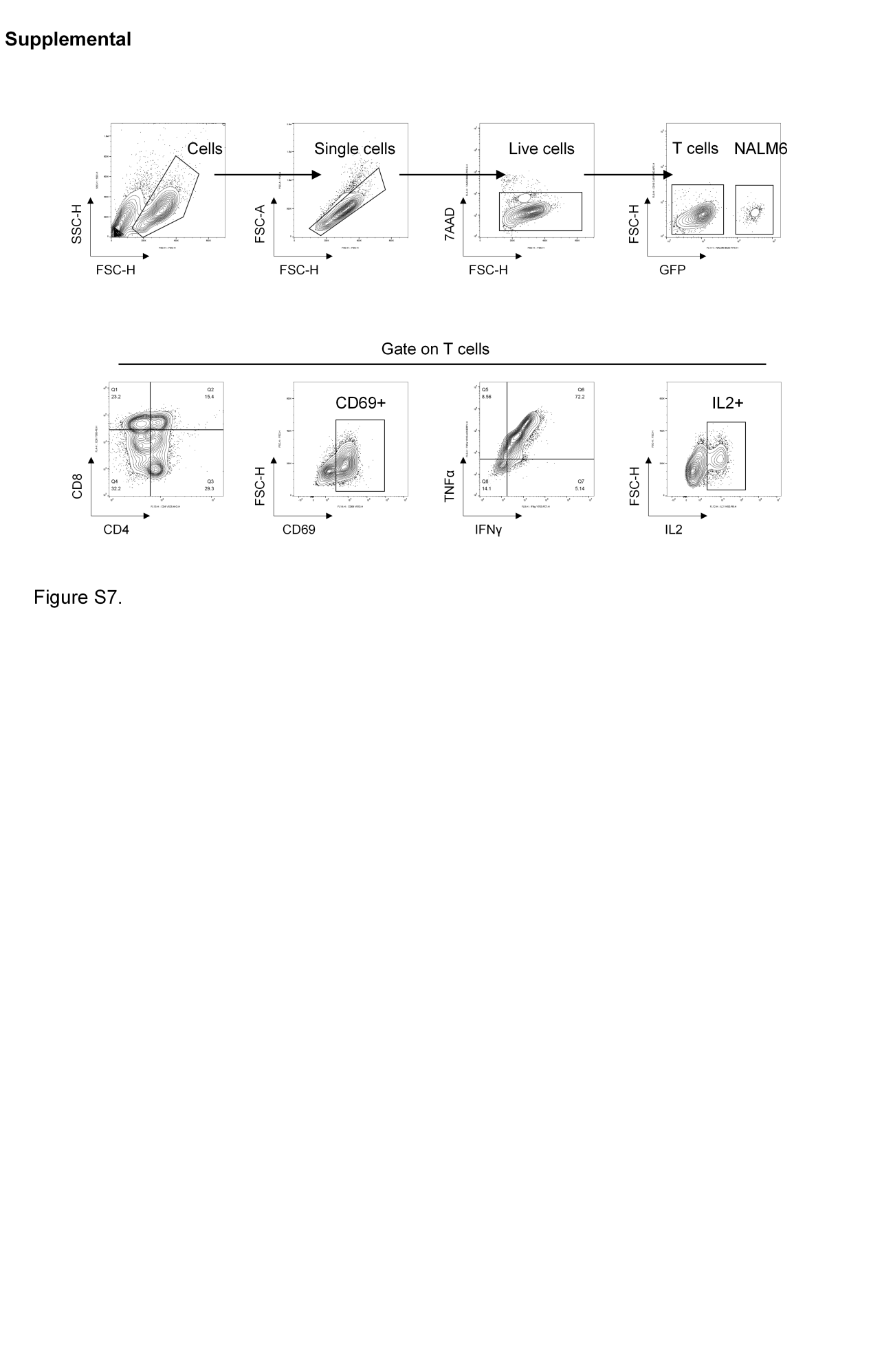


Figure S7.

Cell population were gated based on FSC-H and SSC-H, single cell population were gated based on FSC-H and FSC-A. Live cells were then selected by gating on 7-AAD-negative events. Within the live cell population, NALM6 tumor cells and T cells were distinguished based on GFP expression. All T cell markers and cytokine production were analyzed under the T cell gate. Abbreviations: FSC, forward scatter; SSC side scatter; 7-AAD, 7-aminoactinomycin D.
